# Skin-Interfaced Biosensors and Pilot Studies for Advanced Wireless Physiological Monitoring in Neonatal and Pediatric Intensive Care Units

**DOI:** 10.1038/s41591-020-0792-9

**Published:** 2020-03-11

**Authors:** Ha Uk Chung, Alina Rwei, Aurélie Hourlier-Fargette, Shuai Xu, KunHyuck Lee, Emma C. Dunne, Zhaoqian Xie, Claire Liu, Andrea Carlini, Dong Hyun Kim, Dennis Ryu, Elena Kulikova, Jingyue Cao, Ian C. Odland, Kelsey B. Fields, Brad Hopkins, Anthony Banks, Christopher Ogle, Dominic Grande, Jun Bin Park, Jongwon Kim, Masahiro Irie, Hokyung Jang, JooHee Lee, Yerim Park, Jungwoo Kim, Han Heul Jo, Hyoungjo Hahm, Raudel Avila, Yeshou Xu, Myeong Namkoong, Jean Won Kwak, Emily Suen, Max A. Paulus, Robin J. Kim, Blake V. Parsons, Kelia A. Human, Seung Sik Kim, Manish Patel, William Reuther, Hyun Soo Kim, Sung Hoon Lee, John D. Leedle, Yeojeong Yun, Sarah Rigali, Taeyoung Son, Inhwa Jung, Vinaya R. Soundararajan, Ayelet Ollech, Avani Shukla, Allison Bradley, Molly Schau, Casey M. Rand, Lauren E. Marsillio, Zena L. Harris, Yonggang Huang, Aaron Hamvas, Amy S. Paller, Debra E. Weese-Mayer, Jong Yoon Lee, John A. Rogers

**Affiliations:** 1 Simpson Querrey Institute, Northwestern University, Chicago, IL 60611, USA; 2 Department of Electrical and Computer Engineering, Northwestern University, Evanston, IL 60208, USA; 3 Center for Bio-integrated Electronics, Northwestern University, Evanston, IL 60208, USA; 4 Department of Dermatology, Feinberg School of Medicine, Northwestern University, Chicago, IL 60611, USA; 5 Department of Materials Science and Engineering, Northwestern University, Evanston, IL 60208, USA; 6 Division of Pediatric Autonomic Medicine, Department of Pediatrics, Ann & Robert H. Lurie Children’s Hospital of Chicago, Chicago, IL 60611, USA; 7 State Key Laboratory of Structural Analysis for Industrial Equipment, Department of Engineering Mechanics, Dalian University of Technology, Dalian 116023, China; 8 Department of Biomedical Engineering, Northwestern University, Evanston, IL 60208, USA; 9 Department of Electrical and Computer Engineering, University of Illinois at Urbana-Champaign, Urbana, IL 61801, USA; 10 Sibel Inc., Evanston, IL 60208, USA; 11 Photo-Electronic Hybrids Research Center, Korea Institute of Science and Technology (KIST), Seoul 136-791, South Korea; 12 Department of Mechanical Engineering, Kyung Hee University, Yongin 17104, Republic of Korea; 13 Frederick Seitz Materials Research Laboratory, University of Illinois at Urbana-Champaign, Urbana, IL 61801, USA; 14 Department of Computer Science, University of Illinois at Urbana-Champaign, Urbana, IL 61801, USA; 15 Department of Mechanical Engineering, Northwestern University, Evanston, IL 60208, USA; 16 Department of Civil and Environmental Engineering, Northwestern University, Evanston, IL 60208, USA; 17 Key Laboratory of C&PC Structures of the Ministry of Education, Southeast University, Nanjing 210096, China; 18 Department of Neurobiology, Northwestern University, Evanston, IL 60208, USA; 19 Department of Biology, Northwestern University, Evanston, IL 60208, USA; 20 University of Illinois College of Medicine at Chicago, Chicago, IL 60612, USA; 21 Department of Graphic Design and Industrial Design at North Carolina State University, Raleigh, NC 27695, USA; 22 Department of Pediatrics, Ann & Robert H. Lurie Children’s Hospital of Chicago, Chicago, IL 60611, USA; 23 Division of Neonatology, Department of Pediatrics, Ann & Robert H. Lurie Children’s Hospital of Chicago, Chicago, IL 60611, USA; 24 Stanley Manne Children’s Research Institute, Ann & Robert H. Lurie Children’s Hospital of Chicago, Chicago, IL 60611, USA; 25 Department of Chemistry, Northwestern University, Evanston, IL 60208, USA; 26 Department of Neurological Surgery, Feinberg School of Medicine, Northwestern University, Chicago, IL 60611, USA

## Abstract

Standard of care management in neonatal and pediatric intensive care units (NICUs and PICUs) involve continuous monitoring of vital signs with hard-wired devices that adhere to the skin and, in certain instances, include catheter-loaded pressure sensors that insert into the arteries. These protocols involve risks for complications and impediments to clinical care and skin-to-skin contact between parent and child. Here we present a wireless, non-invasive technology that not only offers measurement equivalency to these management standards but also supports a range of important additional features (without limitations or shortcomings of existing approaches), supported by data from pilot clinical studies in the neonatal intensive care unit (NICU) and pediatric ICU (PICU). The combined capabilities of these platforms extend beyond clinical quality measurements of vital signs (heart rate, respiration rate, temperature and blood oxygenation) to include novel modalities for (1) tracking movements and changes in body orientation, (2) quantifying the physiological benefits of skin-to-skin care (e.g. Kangaroo care) for neonates, (3) capturing acoustic signatures of cardiac activity by directly measuring mechanical vibrations generated through the skin on the chest, (4) recording vocal biomarkers associated with tonality and temporal characteristics of crying impervious to confounding ambient noise, and (5) monitoring a reliable surrogate for systolic blood pressure. The results have potential to significantly enhance the quality of neonatal and pediatric critical care.

 In the United States, over 480,000 critically-ill infants and children enter intensive care units (ICUs) each year. Those less than one year of age suffer from the highest morbidity and mortality rates and therefore require the most intensive care^1,2^. These fragile patients include premature infants who may weigh as little as 500 g (1.1 lbs), one-seventh that of an average term baby^3^. Continuous monitoring of vital signs is essential, yet existing technologies require the use of multiple electrodes and interfaces attached to the skin with adhesive tapes and connected by wires to electronic processing systems that are often tethered to the wall. This system of hardware often frustrates both routine and specialized procedures in clinical care, ranging from therapeutic skin-to-skin contact with parents (i.e. kangaroo care, KC) to feeding, diaper changing and bathing^3^. Furthermore, continuous monitoring of certain vital signs, such as blood pressure, demands additional wired hardware, such as peripheral and umbilical arterial catheters^4^, which can induce other types of complications, including thrombus formation and occlusion, infection (e.g. sepsis), rupture, pseudoaneurysm, bleeding, and death^5–7^. A wireless system capable of non-invasive, continuous, accurate vital sign monitoring has the potential to greatly enhance the effectiveness and safety of neonatal and pediatric critical care^8^.

A recent report by our group^9^ describes a soft, skin-like electronic system designed to address these unmet clinical needs. Evaluation studies in the NICU confirm capabilities for clinically accurate measurements of heart rate (HR), blood oxygenation (SpO_2_), temperature, respiration rate (RR) and pulse wave velocity in the NICU. However, this system is limited by (1) the modest maximum operating distances (~30 cm) supported by near-field communication (NFC) protocols used for power transfer and data communication, (2) the mechanically fragile nature of the ultrathin, compliant mechanics designs, (3) the sufficient, but constrained range of measurement capabilities, and (4) the demand for highly advanced device configurations, capable of fabrication only in specialized facilities with customized tools. The results reported in this manuscript adopt and extend similar principles in soft electronics design, but in mechanically robust, manufacturable systems that rely on Bluetooth technology to circumvent these limitations. These systems include a range of options in operation and power supply that address a broad spectrum of clinical use cases and provider preferences, ranging from modular primary batteries to integrated secondary batteries to wireless power harvesting schemes. These platforms additionally support important modalities in monitoring that lie beyond both the standard of care and the capabilities of our previously reported systems. These include the ability to: (1) monitor movements and changes in body orientation, (2) track and assess the therapeutic effects of KC and other forms of hands-on care, (3) capture acoustic signatures associated with cardiac activity by capturing mechanical vibrations generated through the skin on the chest wall reflective of valvular function (4), record vocal biomarkers associated with tonality and temporal characteristics of crying, and (5) quantify pulse wave dynamics through multiple measurements, as a reliable surrogate for systolic blood pressure.

The ability for this system to provide addition quantitative information on hemodynamic and cardiovascular health states beyond the core vital signs of heart rate, respiratory rate and blood oximetry holds direct relevance to the management of patients in the NICU/PICU^10^. Visualization of heart vibrations, referred to as a seismocardiogram (SCG), is rarely performed in general clinical practice, especially in the NICU/PICU, yet it provides essential information on the mechanical outcomes of myocardial activity, valve motions and other features that are absent from ECG data^11^. Episodic measurements of BP in current clinical practice on neonates and pediatric patients involve miniaturized, but otherwise conventional, BP cuffs that wrap around the limbs, while continuous tracking requires catheter-based pressure sensors (arterial lines) that insert into peripheral arteries. Both procedures, particularly the latter, are invasive and involve multiple risk factors^4^, to an extent that they are used only in limited cases despite the essential utility of the information. The capabilities of the system enable the ability to address aspects of neurological, respiratory, and pathological disorders that are common in premature neonates and can lead to abnormalities in vocalization, range of motion, and posture control^12^. Quantitative, continuous tracking of such behavior offers the potential for early detection of complications associated with birth trauma, brain injury or pain stress^13^. Measurements of movement and physical activity specifically can provide insights into sensorimotor development^12^. These same data can also inform effective methods for neonatal care such as KC, a therapeutic skin-to-skin “treatment”, in which a pediatric patient is held against a parent’s chest in a manner that lowers mortality, stabilizes heart rate, temperature, and respiration rate, and decreases the risks for infection^14,15^.

The following describes the technology platforms, measurement capabilities, clinical effectiveness, and safety through pilot studies on 50 patients across a wide range of ages in both the NICU and the PICU. We present quantitative validation of the full range of capabilities, with comparisons to measurements from FDA-approved instruments, involve continuous monitoring for periods up to 24 hours.

## Results

### Device and System Design

**Fig. 1.**
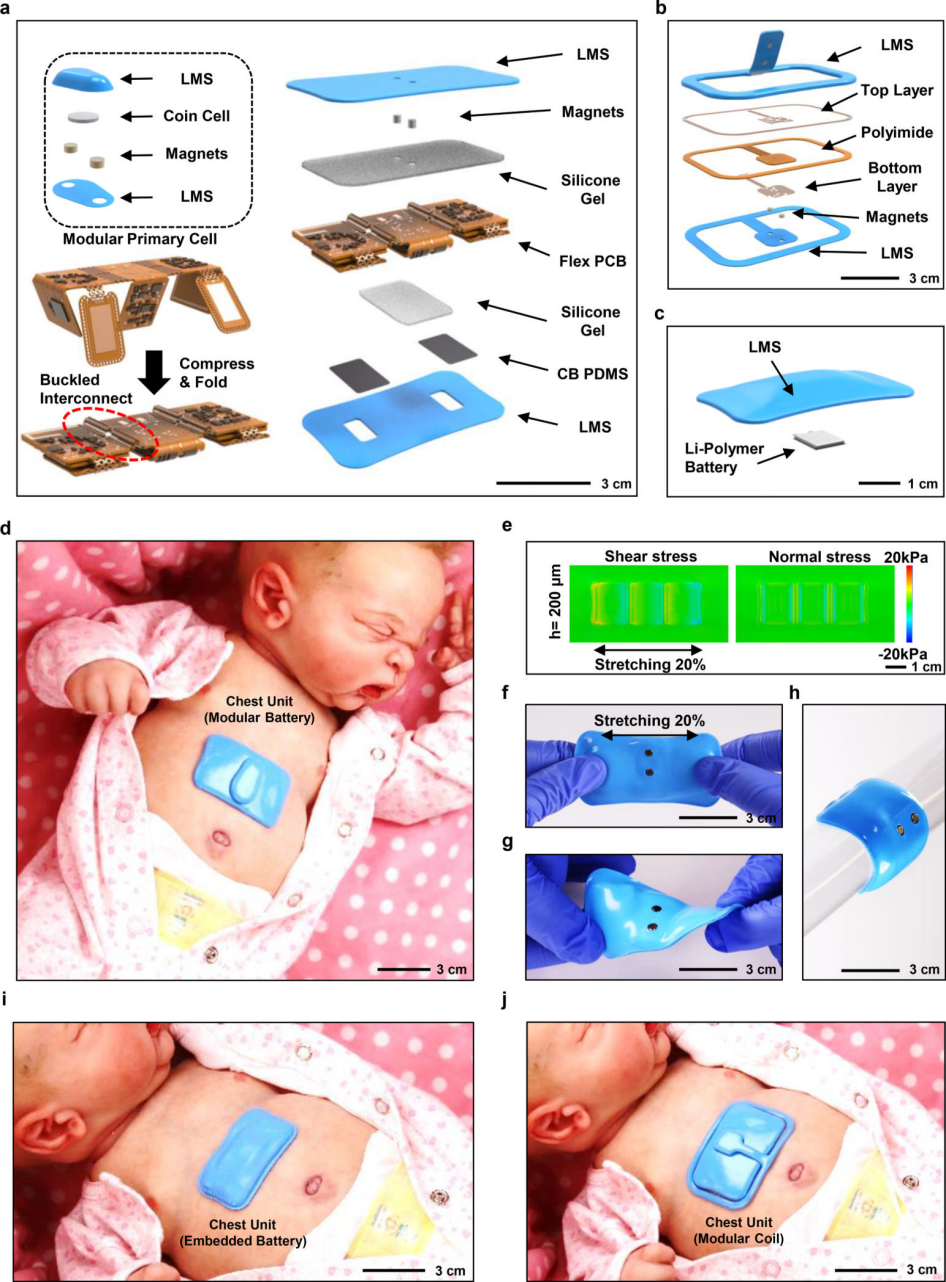
Designs, mechanical characterization results and photographs of a soft, wireless chest unit for physiological monitoring of neonatal and pediatric patients. **a,** Schematic diagram and expanded view illustration of a device with a modular primary battery. The main body consists of buckled serpentine interconnects between islands of electrical components contained within a soft, elastomeric enclosure. The battery interfaces to the system via reversible magnetic coupling. Thin silicone pads establish electrical connections between measurement electrodes and a hydrogel interface to the skin, to yield a completely sealed, waterproof device. LMS stands for low modulus silicone. **b,** Illustration of a detachable wireless power harvesting system. **c,** Illustration of a powering option that involves an integrated, wirelessly rechargeable battery. This option uses a different top layer encapsulation, without the magnets. **d,** Photograph of the chest unit with modular battery on a realistic model of a neonate. **e,** Computed stresses (right: normal; left: shear) at the interface between the skin and a device during uniaxial stretching to a strain of 20%, with a thickness of 200 μm. **f-h,** Photograph of a representative device during stretching (f), twisting (g), and bending (g). **i-j,** Photographs of a device with integrated battery (i) and with a wireless energy harvester (j), both mounted on a model.

Fig. 1a presents an expanded view illustration of one of the two mechanically soft, wireless devices that form the complete system. This example uses a modular battery unit for power supply in a design that allows for gentle placement on the curved skin of the chest (chest unit) via a thin hydrogel coupling layer to record electrocardiograms (ECGs), acoustic signals of vocalization and cardiac/respiratory activity, body orientation and movements, and skin temperature, all enabled by a Bluetooth Low Energy (BLE) system-on-a-chip (SoC) and associated collection of sensors. The overall layout includes a thin, flexible printed circuit board (flex PCB) and mounted components, configured in an open design with serpentine interconnect traces. The construction involves folding of distinct, but connected, platforms as a key step in assembly and packaging (Fig. 1a and Supplementary Fig. 1). Quantitative insights from three-dimensional finite element analysis (FEA) of the system-level mechanics helped to define an optimal distribution of the active components to reduce the lateral dimensions of the device by ~250 %. A pre-compression process in the assembly forms buckled layouts in a serpentine configuration to enhance flexibility and stretchability. An elastomeric enclosure with an inner silicone gel liner (~300 μm thick, ~4 kPa) enhances the device’s softness ensuring compatibility with the fragile skin and highly curved anatomical features of neonates born at the lowest gestational ages. A pair of thin, conductive elements formed using a doped silicone material (carbon black in polydimethylsiloxane, abbreviated as ‘CB PDMS’; bulk resistivity of 4.2 Ω.cm) serve as soft electrical connections to corresponding gold electrodes on the flexible printed circuit board and to conductive hydrogel skin interfaces for ECG measurements. The result is a soft and completely sealed, waterproof system (Supplementary Fig. 2) with applicability across a wide range of settings, focused on but not limited to the NICU and PICU (Supplementary Video S1–3) with robust wireless data transmission and real-time display on the table computer across all locations within a standard patient room within an operational range of 10 meters (Supplementary Fig. 3).

#### Novel Power Management Schemes

The modular battery unit couples to the device mechanically and electrically through pairs of matching sets of embedded magnets (Fig. 1a, inset), thereby: (1) allowing replacement of the battery without removing the device from the patient with the aim to minimize disruptions in clinical care, decrease the burden on clinical staff, and consequently reduce risks of skin injury^16^; (2) enabling removal of the battery to allow autoclave sterilization of device more than 10 cycles without measurable changes in operational characteristics, while the device having embedded battery can be sterilized by use of germicidal disinfecting surface wipes (Sani-cloth, Professional Disposables International, Inc); and (3) mechanically decoupling the battery from the device to improve the bendability and, therefore, the compliance at the skin interface. The magnetic scheme also allows for other options for power supply, not only in choices of battery sizes, shapes and storage capacities (and therefore operational lifetimes), but also in alternative modalities, including battery-free schemes that rely on wireless power transfer (Fig. 1b). As an example of this latter possibility, a magnetically coupled harvesting unit can be configured to receive power from a transmission antenna placed under the bed and designed to operate at a radio frequency of 13.56 MHz with a negligible absorption in biological tissue^17^. Given that a removable battery can act as a swallowing and choking hazard in older infants, the battery can be designed with geometries that are larger than the minimum size requirements for consumer products used by children under the age of three^18^ (Supplementary Fig. 4). Fig. 1c illustrates a third option, in which a wirelessly rechargeable battery (Li-polymer, 45 mAh) lies within the sealed enclosure of the device to eliminate any external connections. The lifetimes for the battery-based platforms depend on the storage capacity of the batteries. The platform with a removable 25 mAh primary cell (CR1216) offers a lifetime of 2 h; a 120 mAh cell (CR1632) offers 8 h. The embedded Li-polymer rechargable battery (45 mAh) can support operation for 30 h. The battery-free configuration provides indefinite lifetime, provided that the device remains in proximity (30 – 50 cm) of a transmission antenna.

#### Sensor Mechanics and Design

The photograph in Fig. 1d features a chest unit deployed on a model of a neonate at an age where the chances of accidental ingestion is remote, with a small (CR1216, volume = 0.2 cm^3^), encapsulated button cell as a modular battery. The serpentine interconnects encapsulated with polyimide (PI), the folded configuration, and the soft enclosure with gel liner lead to a uniaxial elastic stretchability that exceeds ~33 % at the device level, corresponding to a ~500 % stretchability in the interconnects prior to encapsulation in the outer silicone shell (Supplementary Fig. 5 and 6). The gel (~300 μm thick, ~4 kPa modulus) provides strain isolation between the folded islands to reduce the stresses at the skin interface to levels below the thresholds for sensory perception (20 kPa) for uniaxial stretching of up to 20%, a value at the high end of the range expected in practical use (Fig. 1e and Supplementary Fig. 7). The resulting elastic bending radius and equivalent bending stiffness are ~20 mm and ~9.6 Nmm^2^, respectively (Supplementary Fig. 6c). These mechanical characteristics ensure soft, irritation-free skin interfaces, even in cases of extreme curvatures encountered with small babies and/or low gestational ages. The images in Fig. 1f–h illustrate a device stretched, twisted, and bent to levels that exceed ~20%, ~30 °, and ~60 °, respectively. Mounted units with the embedded battery and battery-free designs appear in Fig. 1i and j, respectively.

**Fig. 2.**
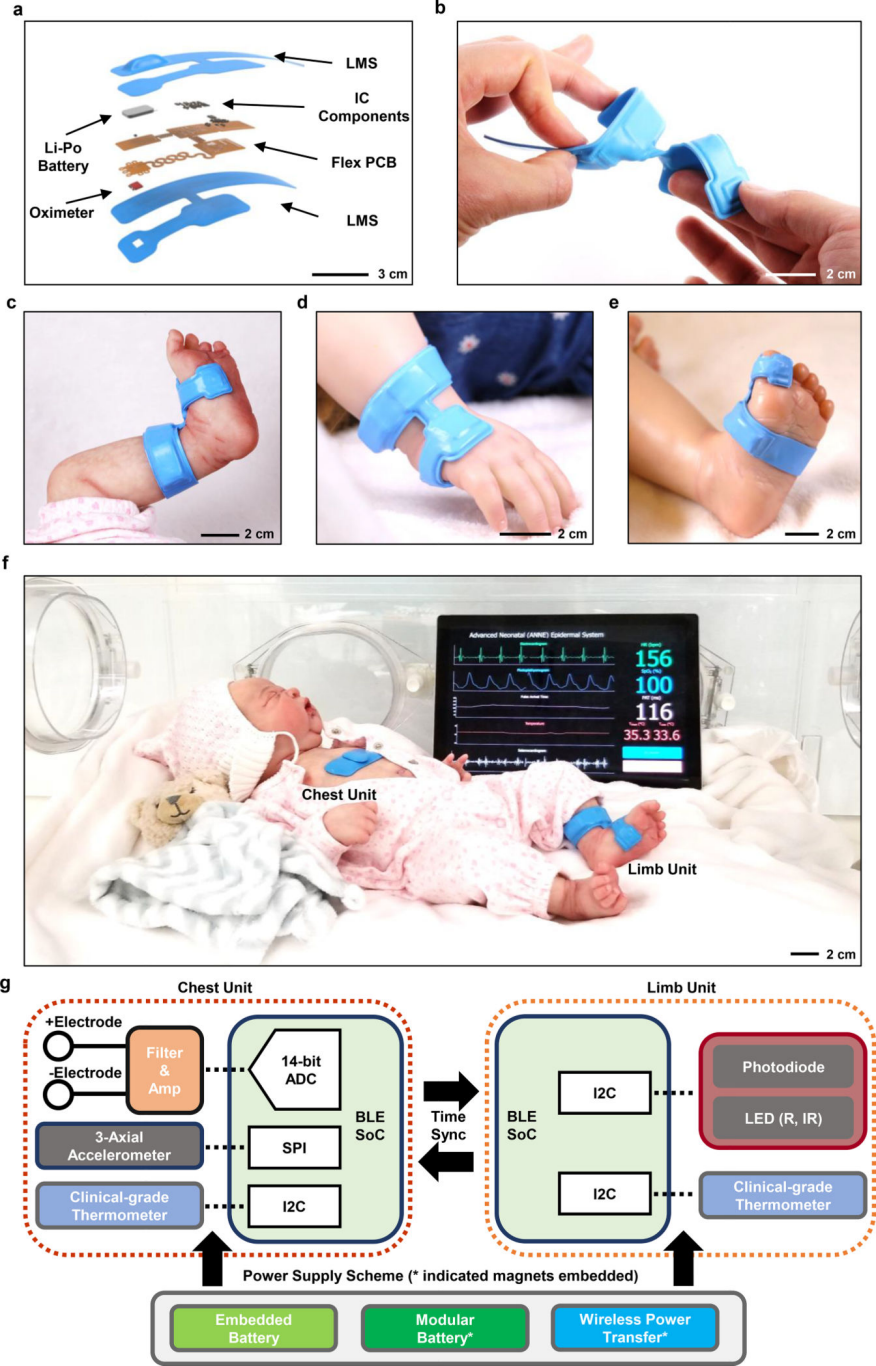
Designs and photographs of a wireless limb unit for physiological monitoring of neonatal and pediatric patients and block diagram of the system operation. **a,** Schematic diagram and expanded view illustration of the limb unit, designed to measure PPG, SpO_2_ and peripheral skin temperature. **b,** Photograph of a device while bent and twisted. **c-e,** Placement of a device on (c) a model of a neonate at the ankle-to-base of the foot, (d) a model of a pediatric patient (2-year-old CA) at the wrist-to-hand, and (e) at the foot-to-toe. **f,** Photograph of the chest and limb unit on a model of a neonate in a NICU isolette, with a tablet computer displaying representative data through a graphical user interface. **g,** Block diagram showing the operational scheme of two time-synchronized devices, with an analog-front-end for ECG processing, 3-axis accelerometer, thermometer IC, and BLE SoC for the chest unit and a pulse oximeter IC, thermometer IC, and BLE SoC for the limb unit. Three different options for power supply appear at the bottom of g.

Fig. 2a summarizes the structure of the other component of the complete system, a device designed to record reflectance-mode photoplethysmograms (PPGs) and skin temperature from peripheral locations. This limb unit features a layout that facilitates wrapping around the foot, palm or toe—this accommodates neonates and pediatric patients of varying ages and anatomies. Fig. 2b highlights the overall design, with umbilical interconnects that can bend to radii as small as ~3.9 mm twist through angles as large as 180° and elastically stretch to uniaxial strains as high as 17% (Supplementary Fig. 8 and 9). The fundamental design features are similar to those of the chest unit, but in configurations that anatomically match different limb interfaces: ankle-to-sole of the foot for neonates in NICU (Fig. 2c) and wrist-to-hand (Fig. 2d) and foot-to-toe (Fig. 2e) for larger, pediatric patients in the PICU. The image in Fig. 2f shows both chest and limb units deployed on a model of a neonate in a NICU isolette, along with a representative illustration of vital signs information displayed on a tablet computer (Supplementary Fig. 10).

Fig. 2g presents a block diagram that summarizes the overall operational scheme. The chest unit includes a wide-bandwidth 3-axial accelerometer (BMI160, Bosch Sensortec), a clinical-grade temperature sensor (MAX30205, Maxim Integrated), and an ECG system that consists of two gold-plated electrodes. The limb unit includes an integrated pulse oximetry module (MAX30101, Maxim Integrated) for measuring dual wavelength PPGs and a temperature sensor (MAX30205, Maxim Integrated). The power management circuit for battery operation uses a voltage regulator to provide supply voltages required for the various components (3.3V or 1.8V). The modular battery-free platform (Fig. 1b) includes an inductive coil tuned to 13.56 MHz, a full-wave rectifier, and a two-stage cascaded voltage regulating unit.

#### Clinical Studies on Neonatal/Pediatric Patients in the Intensive Care Unit

**Fig. 3.**
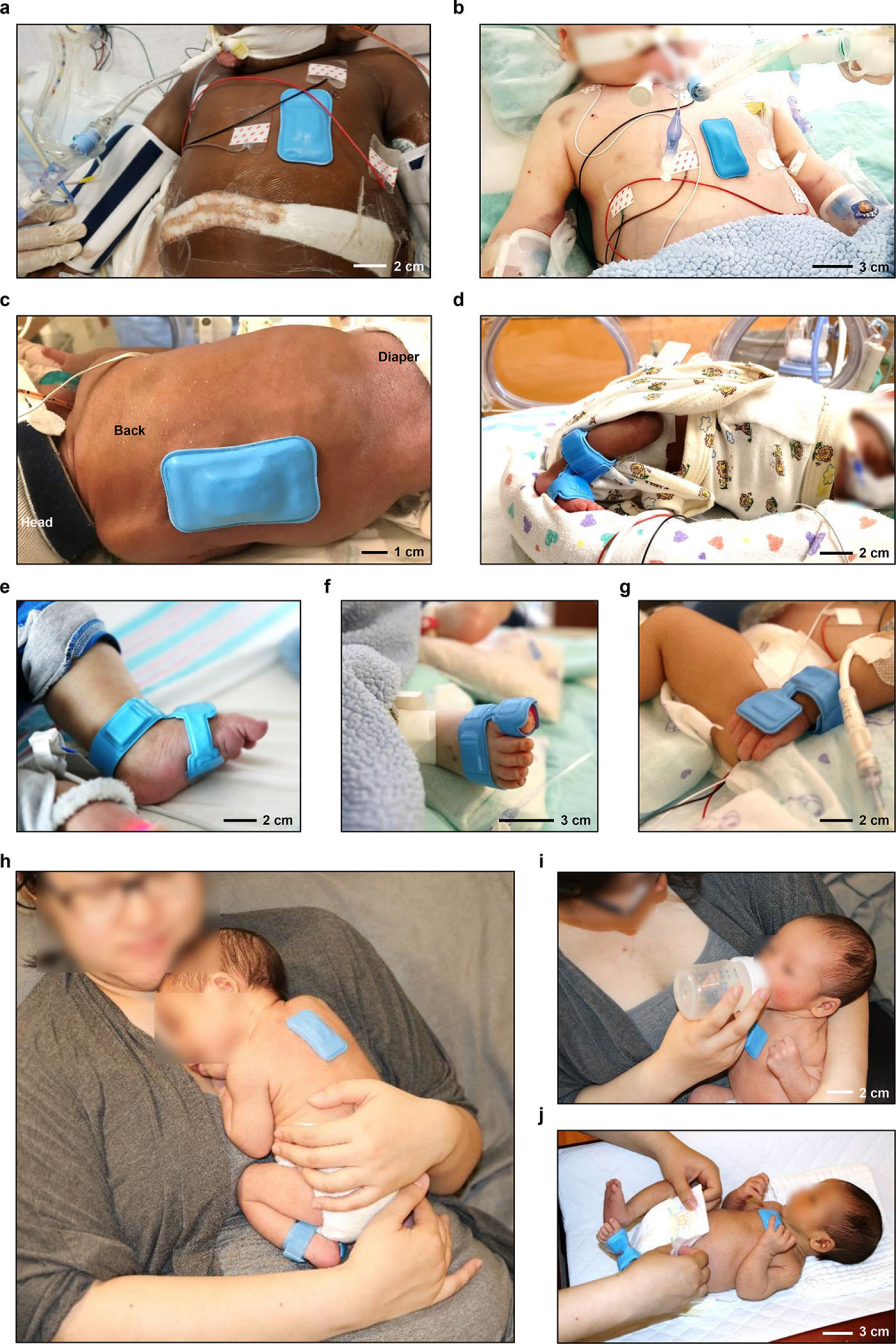
Photographs of wireless wearable devices on neonatal and pediatric patients in the NICU and PICU, respectively, and of parental hands-on care with healthy neonates. **a-c,** Photographs of the chest unit on two different ~2-year-old children (a) and (b) and on a neonatal preterm infant in (c) (27 w GA, 6 w CA) with apnea of prematurity and respiratory failure. Here, the device rests on the back. **d,** Photograph of the limb unit mounted on the ankle-to-foot interface of an infant (27 w GA, 6 w CA) and **e,** another infant (25 w GA, 44 w CA). **f,** Photograph of a similar unit on the foot-to-toe interface of the pediatric patient in (b). **g,** Photograph of a device on the wrist-to-hand interface of a pediatric patient (35 w GA, 6w CA). **h-j,** Photograph of a pair of devices on a 40 w GA healthy neonate during (h) KC, (i) feeding, and (j) hands-on care (diaper change).

The soft mechanical properties and the wireless modes of operation are critically important to effective use on neonatal and pediatric ICU patients, particularly when located at highly curved regions of anatomy on a limited surface area. Fig. 3a,b highlight examples of a chest unit pediatric ICU patients. Fig.3c,d demonstrate the potential for use in extremely premature neonates (27 w gestational age (GA), 6 w chronological age (CA)), including the possibility of mounting the chest unit on the back of the thorax. Fig. 3e–g feature the limb unit at various peripheral locations. Wrapping around the ankle-to-base of the foot is effective for premature neonates^19^, as commonly encountered in the NICU. Other options include mounting around the foot-to-toe (Fig. 3f) or the wrist-to-hand (Fig. 3g), typically most suitable for babies with chronological ages greater than 12 months^20^. These mounting options enhance nearly all aspects of routine and specialized procedures in clinical care, ranging from intimate contact during KC (Fig.3h) and parental holding to feed (Fig. 3i), change diapers (Fig. 3j; Supplementary Fig. 11a), and bathe the infant (Supplementary Fig. 11b).

### Real-time Measurement of Clinical Data in the Neonatal/Pediatric Intensive Care Unit

**Fig. 4.**
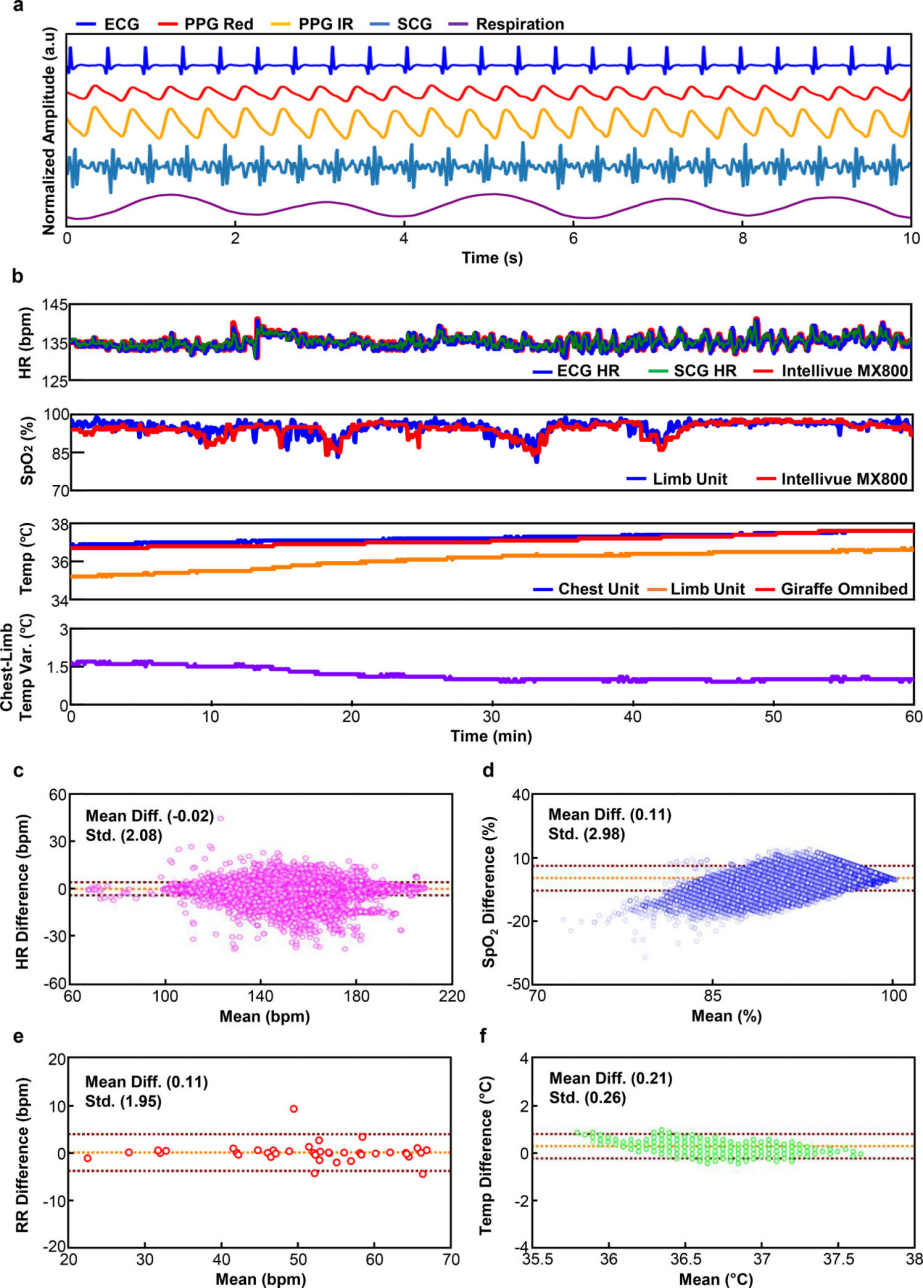
Representative data collected in the NICU and PICU. **a,** Representative ECG, PPG, SCG and respiration waveforms collected from a neonate (29 w GA). **b,** Comparison of HR, SpO_2_, RR, temperature, and temperature gradient between the chest and the foot to standard clinical measurements. **c-f,** Corresponding Bland-Altman plots for (c) HR, (d) SpO_2_, (e) RR, and (f) temperature.

Continous wireless data transmission to a computer system that supports real-time data analytics yields results that can be graphically displayed in an intuitive manner for nurses, doctors and parents. The chest unit measures ECGs and skin temperature, together with a rich range of information that can be inferred from data collected with the high-bandwidth, 3-axis accelerometer, including SCGs, respiration rate and others, with sampling frequencies of 504 Hz (ECG), 0.2 Hz (temperature) and 100 Hz (SCG). The SCG provides information not only on HR, but also on the systolic interval, the pre-ejection period (PEP), and left ventricular ejection time^21^. The limb unit measures PPGs at red (660 nm) and infrared (IR, 880 nm) wavelengths, and skin temperature, sampled at 100 and 0.2 Hz, respectively. Fig. 4a displays representative ECGs, PPGs, SCGs, and chest movements from a neonate with a GA of 29 weeks, captured by continuous wireless streaming to a tablet computer (Surface Pro, Microsoft) using the embedded battery version of the platform through gold electrodes (Fig. 1i).

The streaming raw data from the devices undergoes real-time signal processing on the mobile tablet allowing for dynamic, adaptive vital signs display with negligible time delays (Supplementary Fig. 10 and 12). In many cases, relevant information can be extracted from different, independent data streams. For instance, HR can be obtained from ECG (Supplementary Fig. 13a), PPG, and SCG data separately to yield multiple, redundant estimations. Similarly, RR can be determined, not only from any one of these sources of data, but also from the accelerometry measurements (Supplementary Fig. 13b). Opportunities for exploiting redundancy provided by the full multimodal data suite represent topics of current investigation.

Calculation of peripheral capillary oxygen saturation (SpO_2_) exploits dual color PPGs with algorithms designed to minimize the effects of motion artifacts commonly encountered in the NICU and PICU due to naturally occurring movements (Supplementary Fig. 13c). This platform is effective in detecting rapid temporal changes in frequency, most commonly due to motion artifact, as a simple but effective means to reduce motion artifact effects^22,23^ (Supplementary Fig. 14).

Fig. 4b shows 60 minutes of HR, SpO_2_, and temperature data (see Supplementary Fig. 15 for a magnified plot) obtained on a NICU neonate (GA: 29 wks). These representative data agree well with those captured simultaneously using standard clinical measurements in the intensive care unit (Intellivue MX800, Philips for HR and SpO_2_; Giraffe Omnibed Incubator, GE for temperature; direct physician observation of respiratory rate) with outputs derived from a software license (BedMaster, Anandic Medical Systems). We elected to use direct physician observation of respiratory rate given the known inaccuracies in deriving respiratory rate in critically ill newborns and children from ECG, PPG, or airflow measurements in non-intubated subjects^24^. Quantitative comparisons using the Bland-Altman (BA) method^25^ (Fig. 4c–f) indicate that the mean differences for HR, SpO_2_, and temperature are −0.02 beats per minute, 0.11 %, and 0.21 °C, respectively for a cohort of n = 3 patients. The standard deviations for HR and temperature are 2.08 beats per minute and 0.26 °C respectively. The accuracy root mean square (A_rms_) for SpO_2_ is 2.99%. The mean difference and the standard deviation for RR is 0.11 and 1.95 breaths per minute, respectively for a cohort of n = 6 patients (41 data points). Supplementary Figure 16 presents the global quantitative comparison results particularly for HR and SpO_2_ with over 0.4 million of aggregated data points measured from n = 20 patients. The results for HR and SpO_2_ are within the regulatory guidelines set by the US Food and Drug Administration (FDA), which require errors less than ± 10 % or ± 5 bpm for HR and less than 3.5 % for A_rms_ for reflectance mode SpO_2_^26,27^. FDA guidelines for RR monitors under 21 CFR 870.2375 does not specify requirements in terms of accuracy^28^, but a 510(k) cleared bedside monitoring system (Siemens SC 6000) delivers a target accuracy of ± 3 breath per minute. Further safety testing on additional neonates (n = 50) was conducted to evaluate skin tolerability and ensure negligible heat generation from the sensors operating concomitantly with standard monitoring systems. These results included a diverse range of age groups (23 – 40 wks gestational age and 1 week – 4 yrs chronical age), and ethnicities (16 Caucasian, 24 Hispanic/Latino, and 10 Black/African American) (Supplemental Table 1). Thermal safety testing demonstrated no heat generation from either device (Supplemental Figure 17) in a subset of 3 patients for periods of 24 h, selected to aligns with clinically relevant time scales between cleaning and bathing events. Finally, no skin adverse events were noted in all 50 subjects as graded by the Neonatal Skin Condition Score (NSCS)^29^ (Supplementary Table 1).

### Time-synchronized Bi-nodal Communication for Non-invasive Blood Pressure Monitoring

**Fig. 5.**
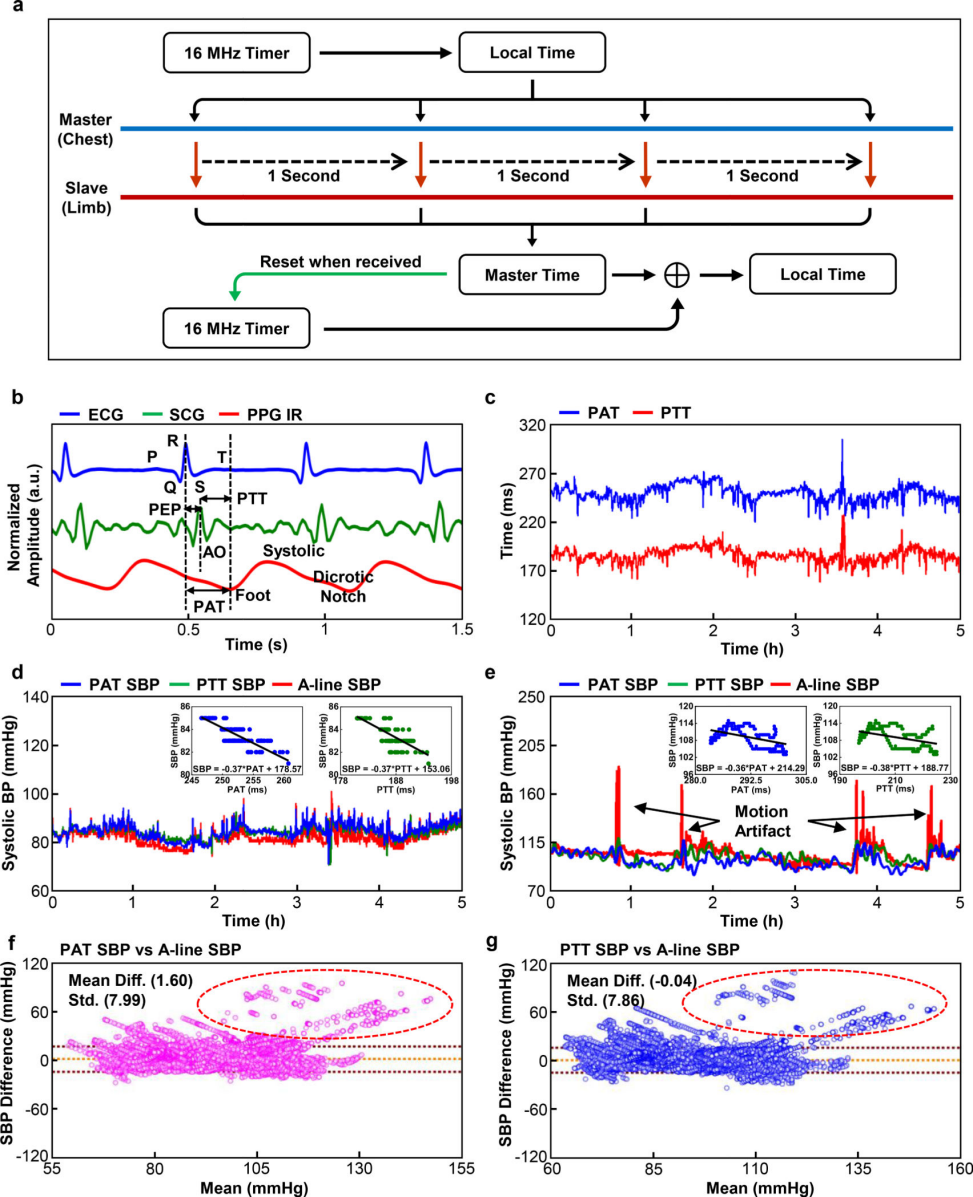
Time-synchronized operation of chest and limb units for measurements of systolic blood pressure, with comparison to arterial line data collected in the PICU on pediatric patients. **a,** Block diagram of the scheme for time-synchronization. **b,** Definition of pulse arrival time (PAT) and pulse transit time (PTT), as derived from ECG, SCG, and PPG waveforms. PEP and AO stands for pre-ejection period and aorta opening, respectively. **c,** Representative PAT and PTT data from a pediatric patient in the PICU during a study over 5 h. **d**, Results of SPB determined with PAT and PTT and with an arterial line (A-line) for an infant (34 w GA, 40 w CA) and **e,** another infant (40 w GA, 50 w CA). **f,g,** Bland-Altman plot for PAT-derived SBP (n = 5) and Bland-Altman plot for PTT-derived SBP (n = 5), respectively. Data points in the red circle indicates the comparison result at the incidents of motion artifact in 5e.

Pulse arrival time (PAT) and pulse transit time (PTT) are two related but distinct measures with established correlations to systolic BP (SBP)^30^. The PAT, calculated from the time difference between the R-peak of ECGs on the chest unit and valley regions of the PPGs on the limb unit, represents the time delay of the pulse pressure wave to travel from the aorta to peripheral limb location at each cardiac cycle. Some studies suggest that the exclusion of the PEP from the PAT may improve correlation with SBP^31,32^. PTT, calculated from the peak-to-foot time delay between the SCG and PPG waveforms (Fig. 5b), achieves this exclusion by capturing the residual peak when the aorta valve opens. Ultimately, both PAT and PTT depend on vascular system geometry, elasticity, SBP, and other factors^30,33,34^. Extensive studies on adult subjects establish calibrated correlations between PAT, PTT and SBP using both empirical and theoretical models^30^, some of which are clinically approved for monitoring in certain scenarios (e.g. Sotera ViSi Mobile® System). Few studies report the correlation of PAT with SBP in infants, mainly in the context of sleep studies and as screening method rather than a core clinical tool.^35,36^ None report measurements of PTT in this critical care population.

This design integrates synchronous operation of the chest and limb devices, enabling measurements of PAT and PTT for each cardiac cycle. To ensure timing accuracy, once every second the chest unit transmits its 16 MHz local clock information to the limb unit, as shown in Fig. 5a. The result eliminates timing drift to enable a synchronization accuracy of greater than 1 ms, on average, and a standard deviation of 3.6 ms over a continuous, 24 h period of operation (Supplementary Fig. 12). This scheme requires an additional current consumption of ~0.2 mA compared to the standard mode of operation. The timing interval of one second provides a tradeoff between power consumption and timing accuracy, given that the measured time delays of interest here are typically > 100 ms. The proportional model^30^ derives the linear relationship of PAT and PTT data to SBP, shown in the equation 1, in which PT can represent either PAT or PTT

(1) $$SBP\;=-a\left(PT\right)\;+b$$

Calculation of coefficients in the equation involves the linear regression of PAT and PTT data to 5 min of SBP data measured using an A-line, which serves as an initial calibration. Figs. 5c–e show PAT and PTT data and their linear relationship to SBP collected simultaneously using an A-line from two different infants in the PICU (40 w CA and 50 w CA, respectively). The demonstration here of exclusion of PEP in the form of PTT is the first reported in NICU/PICU. Accelerometry data of a chest unit (Supplementary Fig. 18) shows the correlated behavior of the overshoot of the A-line data in Fig. 5e with motion artifact. Such modality presents the opportunity to measure the signal quality index to determine the reliability of data at the incidents of movement to derive more reliable SBP output. The results show strong agreement throughout the measurement period (Fig. 5d, e and Supplementary Fig. 19–22). The mean differences of 1.60 and −0.04 mmHg and the standard deviations of 7.99 and 7.86 mmHg (Fig. 5f, g) for PAT and PTT-derived SBP values, respectively, indicate their statistical validity (n = 5). The results are within the ANSI/AAMI SP10 standard, which requires the mean differences and standard deviations of <5 mmHg and <8 mmHg, respectively. Supplementary Fig. 23 summarizes the effects of calibration for the data of Fig. 5d.

### Advanced Use Cases: Kangaroo Care and Cry Analysis

**Fig. 6.**
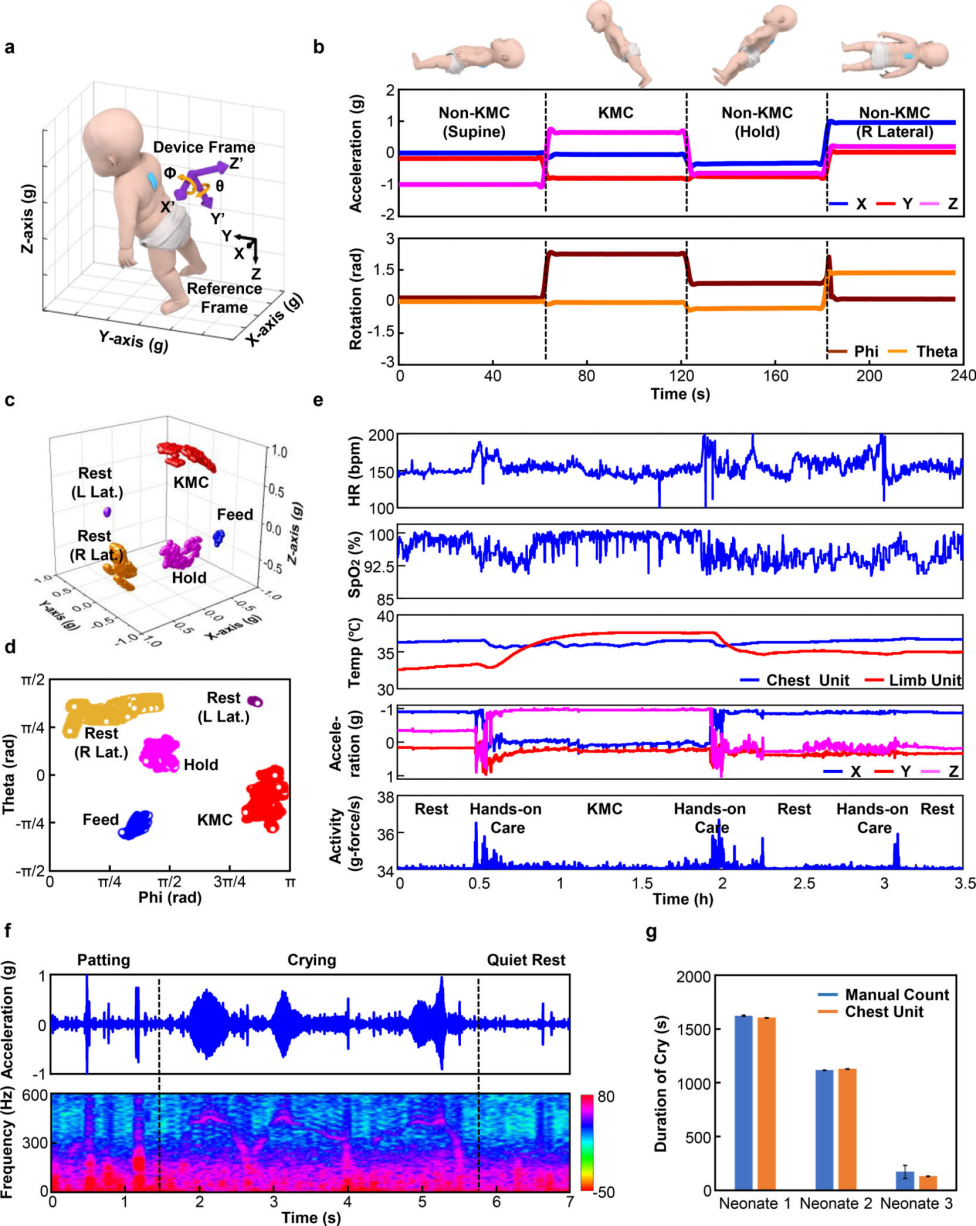
Advanced monitoring modalities based on measurements of orientation, activity and vibratory motions. **a,** Definition of device axes and rotation angle between the device and reference frames. **b,** Orientation data extracted from low bandpass filtering of accelerometry data collected from the chest unit and derived rotation angles for various scenarios: resting in supine and right lateral positions, non-KC and KC holding. **c,** Filtered accelerometry data and **d,** rotational angles for neonates in various body positions in the NICU (n = 3). **e,** Representative HR, SpO_2_, chest and peripheral limb temperature, orientation and activity data defined as the root-mean-square of the 3-axes acceleration values between 1 and 10 Hz before, during, and after KC with a premature neonate (31 w GA). **f,** Raw accelerometry signal (top) and spectrogram of time-frequency signal (bottom) during crying and non-crying events from a neonate (37 w GA, large-for-gestational-age) with feeding difficulties. **g,** Comparison of cry duration determined with the device and by manual recording from neonates (n = 3), with a total of 11 cry events. The resolution of cry duration from our device was 0.2s, whereas manual count ranged from one second to one minute, dependent on the recorder. Error bars were defined as half of the resolution of the measurement.

In addition to SCG and PTT, several additional important modes of operation follow from further use of data from the high-bandwidth 3-axis accelerometer. Examples include motion/movement (including tracking KC and infant holding), and measuring vocal biomarkers such as tonality, dynamics and frequency of crying. According to guidelines from the World Health Organization (WHO), KC involves holding the neonate in an upright position on the parent’s chest, with the neonate’s abdomen placed at the level of the parent’s epigastrium, and the neonate’s head turned to one side to allow eye contact with the parent^37^. This body position, which can be precisely and continuously monitored using low pass filtered (0–0.1 Hz) data from the accelerometer of the chest unit, is distinct from those that occur during most other activities and forms of care. Fig. 6a presents the device and reference coordinate frames and their relative orientations. Here, phi and theta correspond to rotations around the x- and y-axis, respectively, consistent with the right-hand rule. Fig. 6b demonstrates measurements of core body orientation using data from a chest unit placed on the back of a neonate. Here, a stationary hold in the KC position yields phi and theta angles of 2 ~ 3 rad and −0.5 ~ 0 rad with respect to the reference frame, respectively. Data collected for the cases of supine, horizontal and right lateral orientations are each significantly different from the KC position (P-values < 10^−5^ for all positions compared with KC) in terms of rotational angle. Figs. 6c and d provide three-dimensional representations and angles obtained in the NICU during a KC session. The KC events correspond to 2.85 ± 0.10 rad, −0.29 ± 0.28 rad (data are mean ± std for 2.4 h) in phi and theta respectively. Comparisons between resting (right and left lateral position), holding, feeding, and KC events in this clinical environment each show significant differences in 3-axes acceleration and rotational angle (P-values < 10^−5^, n = 3).

Fig. 6e highlights results of HR, SpO_2_, central and peripheral skin temperature, along with a measurement of activity derived from the accelerometry data before, during, and after the KC study, including removal and return of the neonate to the crib. Here, activity corresponds to the root mean square value of 3-axis accelerometry data after bandpass filtering between 1 and 10 Hz^38^. The data show that skin-to-skin contact during KC produced a pronounced, gradual increase in the peripheral skin temperature, consistent with expectation and as demonstrated in previous studies^39^. The mean activity levels during rest and KC events are 0.07 ± 0.02 g/s, while during hands-on care these values are 0.24 ± 0.05 g/s (data are mean ± std for 3 neonates, total 8 hours of KC/rest and 75 min of hands-on care). These data have potential to provide a quantitative indicator to help minimize the disturbance of neonates during various forms of care, and, therefore, risks of hypopnea, apnea, and oxygen desaturation^40^. Current work seeks to explore this opportunity and to establish methods to use the full set of measurement results to provide feedback on the timing and techniques of KC, particularly in sessions extending beyond 4 hours, in which the impact on physiological parameters are expected to be enhanced.

In addition to activity, orientation and SCG, the accelerometer also yields information on vocal biomarkers generally, and crying in particular, via analysis of the high frequency components of the data. Cry analysis can serve as a non-invasive method to analyze the neurophysiological state, often influenced by birth trauma, brain injury or pain stress^13^. Crying captured by measurements with microphones^41^ are easily confounded by ambient sounds in the environment, a particular challenge in NICU and PICU settings. The accelerometer, by contrast, responds only to mechanical vibratory motions of the chest, and is nearly completely unaffected by ambient noise^42^. Fig. 6f shows typical data (top) and the time-frequency signal (bottom) captured at a sampling rate of 1600 Hz from a representative neonate. The signals associated with crying have distinctive frequencies (typically between 400 and 500 Hz, with strong harmonics), well separated from other physiological effects such as cardiac activity (1 – 50 Hz) and muscle tremors (< 20 Hz)^43–45^ or from various operations in care such as patting, rubbing or stroking (see Supplementary Figs. 24a–c). Fig. 6g summarizes 11 crying events measured in this manner, and in a process of manual recording at the bedside (n = 3 infants). The duration of events captured using these two approaches show an average difference of −3.9 ± 13.9 s (data are mean ± std for 11 cry events) (Supplementary Fig.24d). The fundamental frequency of 410.7 ± 47.9 (Supplementary Fig.24e) is consistent with published results^46^.

## Discussion

Pilot studies on patients in NICU and PICU demonstrate the feasibility of a pair of soft, skin-interfaced wireless devices to capture HR, SpO_2_, RR, as well as core and peripheral temperature with high levels of reliability and accuracy as compared with clinical standard monitoring systems that use conventional, hard-wired interfaces. In fact, the data indicate that in many cases the wireless operation and the gentle, mechanically stable measurement interfaces reduce the magnitude and prevalence of noise artifacts associated with motions and other parasitic effects, compared to wired systems. At the same time, the comparisons generated in this study reflect real-world calculations of vital sings in patients undergoing clinical care, feeding, mediacl imaging, and other porcedures. Thus, inherent in this comparision is motion artifact and noise that is processed and analyzed distinctly between our experimental system and the clinical compartor. In instances of low SpO_2_ (< 90%), the mean disagreement is higher than the overall comparison. We believe that this likely reflects different SpO_2_ de-noising algorithms rather a true different in measurement—in our patient cohort, subjects are rarely hypoxemic for a sustained period of time without medical intervention such as supplemental oxygen. In addition to these basic vital signs, time synchronization techniques yield data that serve as promising surrogates of SBP, thereby offering the potential to bypass the use of cuffs for episodic measurements^47^ and arterial lines for continuous tracking^48^. Predicate results on adults and pediatric populations lend confidence in the findings presented here, as the first measurements that exploit accelerometer-based PTT on patients in the NICU and PICU. The device designs and the simplicity of the user interface suggest opportunities for deployment outside of traditional NICU/PICU facilities, into the developing world and even into the home. The availability of continuous, high quality digital data streams in these and other contexts suggest opportunities for use of advanced analytics to extend the range of utility in clinical and remote care.

Another important outcome of the work is in demonstrated capabilities for capturing advanced and unusual physiological signals such as SCG, body orientation, activity and vocal biomarkers. Cardiac monitoring with SCG yields important data to complement those associated with ECG, with enhanced utility in early detection of cardiac complications^49^. Although SCG measurements are reported on adult populations^50^, their use in routine clinical practice is rare and is absent in neonatal and/or pediatric contexts due, at least partly, to the high degrees of curvature of the chest^51^ and the fragility of the skin surface^52,53^. The same data streams yield, through digital filtering techniques, information on body orientation and activity, which are relevant to identifying and quantifying KC, feeding, holding, resting, patting, and potentially sleep patterns. Quantifying such measures has potential to provide insight into the role these activities have on physiologic stability, neurodevelopmental, and other short and long term outcomes.^54^ The collective suite of measurements may allow optimization and enhancements in care, in which vital signs and other parameters can serve as guiding signatures of efficacy. Here, as well as in traditional vital signs monitoring, advanced analytics, including methods such as machine learning, may be very powerful. Such techniques could offer particular value in the analysis of neonatal cry, as a rich source of information that represents the main method for neonates to communicate distress^55^. Studies in controlled settings using microphone recordings indicate that cry patterns reflect neurodevelopment and physiological status, with potential relevance to the detection of sudden infant death syndrome^56^, asphyxia, congenital heart diseases, and respiratory distress syndrome^57^. The platforms introduced here eliminate difficulties associated with ambient sounds in the noisy environments of the NICU and PICU, thereby creating an opportunity to exploit this relatively underexplored, yet rich source of information in settings of practical interest^41^.

The robustness of the platforms, the options in power supply, the sealed/waterproof construction, the soft mechanics, the skin-safe adhesive interfaces with no instances of skin tearing or dermatitis associated with the devices, the compatibility with established sterilization techniques, the re-usability of the devices, and the alignment of the constituent components, materials and designs with advanced manufacturing practice suggest broad deployability. The outcomes have the potential to enhance the quality and breadth of information for physicians, nurses and parents responsible for the care of neonatal/pediatric patients. A growing base of multilateral physiological data, most notably continuous heart activity, respiration, temperature, blood pressure, motion, body orientation, and vocal biomarkers, coupled with advanced learning algorithms, may facilitate early diagnosis of many common complications in these populations, including seizures, and apnea, upon extensive collection and analysis of data from relevant clinical studies^58^. The core technology, beyond neonatal and pediatric critical care, has clear applications in post-acute monitoring, outpatient or home settings, trauma situations, and low-resource environments through simultaneous capturing of critical vital signs and novel digital biomarkers.

## Online Methods

### Fabrication and assembly of the chest and limb devices

Fabrication involved soldering electronic components onto flexible printed circuit boards patterned using a laser ablation process. Embedding the assembled and folded system into a soft silicone enclosure completed the process. For the chest unit with modular options in power supply, films of a soft silicone material (Silbione RTV 4420; Part A & Part B, mixed with 5% of Silc-Pig silicone opaque dye) formed by spin-cast at 250 rpm and thermally curing (100 °C in an oven for 20 min) on glass slides served as top and a bottom layers for the encapsulation process. Curing of both layers involved heating to 100 °C in an oven for 20 minutes. A cutting process with a CO_2_ laser (ULS) defined openings for the ECG electrodes on the bottom layer and for magnets on the top layer. A silicone-based adhesive (3M 96042) bonded the electronics to the bottom layer. Pre-compression of the serpentines during this step ensured high levels of stretchability, with associated enhancements in the bendability. A silicone gel (Ecoflex, Smooth-On) cured at 100°C for 20 min provided a soft, strain-isolating interface layer both below (center part) and above (whole coverage) the electronics. Bonding an overlayer of Silbione finalized the encapsulation process. A drop-casting technique formed coatings of Silbione on top of the various modules for power supply.

Fabrication of the integrated secondary battery version of the device exploited a related encapsulation process, but designed to yield an enclosed air-pocket design as a strain insulation layer to minimize the mechanical load associated with the battery. Here, Silbione cast in a machined aluminum mold served as a top capping layer. A film of this same material, formed as previously described, served as the bottom seal against the perimeter region of the shell to complete the enclosure.

An analogous process defined the encapsulating enclosure for the limb unit, with transparent regions at the location of the LED module for PPG measurements. For all devices, a final laser cutting step yielded a smooth, clean perimeter.

### Preparation of soft, integrated electrodes of PDMS doped with carbon black (CB-PDMS)

The formulation involved the addition of 4.5 g of carbon black to 15.0 g of a silicone prepolymer (Sylgard 184 base) in a 200 mL round-bottom flask containing n-hexanes (100 mL) and stirred vigorously with a stir bar for 10 min at room temperature. Addition of 1.5 g of silicone curing agent (Sylgard 184 curing agent) pre-diluted in 1 mL hexane with continuous stirring for 2–3 min induced polymerization. Rotary evaporation at 40 °C led to simultaneous rapid removal of solvent and degassing of the polymer to yield a smooth paste. Uncured CB-PDMS, spread with a flat edge onto glass slides containing level guides coated with mold release spray (Ease Release 200, Mann Release Technologies), yielded thin solid films of CB-PDMS (250 μm thickness) after curing overnight in an oven at 70 °C. Electrode pads, cut with a CO_2_ laser to lateral geometries larger by 2 mm along all edges of the openings for the ECG electrodes on the bottom surfaces of the chest unit, provided overlapping regions for bonding. Treatment of both elastomeric surfaces with a corona gun (BD-20A High Frequency Generator, Electro-Technic Products, Inc.) for 40 s, immediately followed by pressure induced lamination (15 s) and overnight curing at 70 °C in an oven yielded excellent adhesion. A double-sided conductive tape (3M 9719) bonded the CB-PDMS pads to the Au electrodes on the flexible printed circuit board.

### Water Immersion Tests of the Encapsulation Structure

Tests of permeation used platforms with the electronic components replaced with a dessicant (Drierite) (n = 4). Studies involved daily gravimetric measurements following continuous immersion in 1x DPBS (Dulbecco’s Phosphate Buffered Saline) at 37 °C. A rapid increase in device weight (>1000 mg in 24 h) at ~19–28 days followed from partial delamination of the perimeter seal between the top and bottom Silbione layers, as opposed to the seal between the CB-PDMS and Silbione. Additional tests with a functional chest unit continuously immersed in 1x DPBS at 70 °C, demonstrated stable operation, evaluated daily, for 18 days.

### Quantifying Time Synchronous Operation

Characterization of time synchronization used a two-channel function generator to provide a pair of periodic signals (20 ms 3.5V square pulses separated by 1s) with a controlled time delay between the two. Connecting one channel to the ECG module and the other to a red LED placed on top of the PPG module, yielded data that validated synchronization to a mean delay of less than 1 ms and a standard deviation of 3.6 ms (Supplementary Fig. 10).

### Testing of Compatibility with Autoclave Sterilization

The tests focused on a chest unit with a modular primary battery and a Heidolph Tuttnauer 3545E Autoclave Sterilizer (Electronic Model AE-K). The process involved a temperature ramp to 121°C, a sterilization time of 15 min, and a drying time of 60 min, performed using a device with the battery removed. Functional tests before and after sterilization revealed no change in performance.

### Characterizing the Temperature Sensor

Measurements of the accuracy of the temperature sensor involved immersion in a water bath, heated to 42 °C and then cooled to room temperature, with simultaneous measurements using a reference thermometer (Fisherbrand™ 13202376, Fisher Scientific) as a standard.

### Clinical Testing

The research protocol was approved by the Ann & Robert H. Lurie Children’s Hospital of Chicago and Northwestern University’s Institutional Review Board (STU00202449) and registered on ClinicalTrials.gov (NCT02865070). IRB 2018–1668, approved by the Institutional Review Board of the Ann & Robert H. Lurie Children’s Hospital of Chicago in July 2018, was also used in this study. After informed consent from at least one parent for all participants, the experimental sensors were placed on the chest and limb (foot or hand) by trained research staff. The sensors were placed in a way as to not disrupt any of the existing clinical monitoring equipment. No skin preparation was conducted prior to sensor placement or with sensor removal. The protocol enabled collection times of up to 24 hours. However, medical procedures (e.g. surgery) or imaging required removal of the sensors. Upon removal of the sensors, a board-certified dermatologist evaluated the underlying skin for evidence of irritation, redness, or erosions. Data were transmitted, collected, and stored for further data analysis on a tablet PC (Surface Pro 4, Microsoft) placed out of view from parents and clinical staff. For Kangaroo Care study, the parent is dressed with an open-front hospital gown and the nursing staff facilitates the gentle tranfer of the infant to the parent. The infant is rested on the parent’s chest in an upright position, head tilted to the side and prone with their legs and arms flexed. This position allows th the parent to provide skin-to-skin contact with the infant. The parent is seated for ~ 1h with the infant resting on the parent’s chest., thereby completing Kangaroo Care. All subjects in the Northwestern Prentice Women’s Hospital and Lurie Children’s Hospital admitted to the neonatal intensive care unit and pediatric intensive care unit were eligible regardless of gestational age.

The authors affirm that human research participants provided written informed consent, for publication of the images in Fig. 3, Supplementary Fig. 11, Supplementary Video S1.

### Data Analysis and Algorithms – KC and Cry Analysis

KC analysis relied on accelerometer measurements captured at a sampling rate of 100 Hz. Calibration involved aligning the x-, y-, and z-axes of the device with the gravity vector. Signal processing used a Butterworth low pass filter (3^rd^ order) with a cutoff frequency at 0.1 Hz. Simple trigonometry defined the orientation angle from the acceleration values. Results plotted in three dimensions were correlated to manually recorded body positions. Processing the acceleration signal through a Butterworth bandpass filter (3^rd^ order) between 1–10 Hz, followed by computation of the root-mean-square of the acceleration values along the x-, y-, and z- axes yielded a metric for neonatal activity level, determined each second.

Recording vibratory signatures of vocalization, including crying, involved operation of the accelerometer at a sampling rate of 1600 Hz. Signal processing used a Butterworth high pass filter (3^rd^ order) with a 20 Hz cutoff frequency. Fast Fourier transforms yielded power spectra on time segments with durations of 200 ms. Cry events correspond to spectra with significant peaks between 350 Hz and 500 Hz, with exclusion of harmonics from lower frequency signals (such as those due to patting).

Statistical analysis used a one-way Multivariate Analysis of Variance (MANOVA) via MATLAB, with an assumption that data points for each group are normally distributed. P-value < 0.05 was considered significant.

## Supplementary Material

Chung_NatureMed_SupplMat

Chung_SupplVid1

Chung_SupplVid2

Chung_SupplVid3

Chung_SupplVid4

Chung_SourceData_Fig4

Chung_SourceData_Fig5

Chung_SourceData_Fig6

Chung_SourceData_ExtDataFig2

Chung_SourceData_ExtDataFig4

Chung_SourceData_ExtDataFig5

Chung_SourceData_ExtDataFig7

Chung_SourceData_ExtDataFig8

Chung_SourceData_ExtDataFig9

Chung_SourceData_ExtDataFig10
