# Supplementary material for: Skin-Interfaced Biosensors and Pilot Studies for Advanced Wireless Physiological Monitoring in Neonatal and Pediatric Intensive Care Units
Source: Nat Med. Author manuscript; Available in PMC 2020 Jun 25. (PMC7315772; doi:10.1038/s41591-020-0792-9)
Supplement: Chung_NatureMed_SupplMat [file NIHMS1598235-supplement-Chung_NatureMed_SupplMat.docx]

**Skin-Interfaced Biosensors and Pilot Studies for Advanced Wireless Physiological Monitoring in Neonatal and Pediatric Intensive Care Units**

Ha Uk Chung, Alina Rwei, Aurélie Hourlier-Fargette, Shuai Xu, KunHyuck Lee, Emma C. Dunne, Zhaoqian Xie, Claire Liu, Andrea Carlini, Dong Hyun Kim, Dennis Ryu, Elena Kulikova, Jingyue Cao, Ian C. Odland, Kelsey B. Fields, Brad Hopkins, Anthony Banks, Christopher Ogle, Dominic Grande, Jun Bin Park, Jongwon Kim, Masahiro Irie, Hokyung Jang, JooHee Lee, Yerim Park, Jungwoo Kim, Han Heul Jo, Hyoungjo Hahm, Raudel Avila, Yeshou Xu, Myeong Namkoong, Jean Won Kwak, Emily Suen, Max A. Paulus, Robin J. Kim, Blake V. Parsons, Kelia A. Human, Seung Sik Kim, Manish Patel, William Reuther, Hyun Soo Kim, Sung Hoon Lee, John D. Leedle, Yeojeong Yun, Sarah Rigali, Taeyoung Son, Inhwa Jung, Vinaya R. Soundararajan, Ayelet Ollech, Avani Shukla, Allison Bradley, Molly Schau, Casey M. Rand, Lauren E. Marsillio, Zena L. Harris, Yonggang Huang, Aaron Hamvas, Amy S. Paller, Debra E., Weese-Mayer, Jong Yoon Lee, John A. Rogers

**This PDF file includes:**

Materials and Methods

Fig. S1 to S25

Table S1

**Other Supplementary Material for this manuscript include the following:**

Table S2 – Vital Signs Data

Table S3 – Blood Pressure Analysis

Video S1 – Adult Demo

Video S2 – Demo Waterproof

Video S3 – Real-time streaming in the actual experiment in PICU (de-identified)

Video S5 – Cry Pattern

Materials and Methods

*Flexible printed circuit board fabrication and assembly*

Flexible printed circuit board (FPCB) was designed using Eagle CAD Version 9 (Autodesk) for sensors. Generated gerber files were then used by an ISO 9001-registered PCB manufacturer (Rush PCB Inc.) to fabricate FPCB. Fully assembly of components was also performed by the same vendor. A summary of bill of materials used for assembly is provided below.

- Chest Sensor
- Passive components: Capacitors, Inductors, Resistors; 0201 to 0402 footprint)
- Power management units: Bq25120a (Texas Instruments), TPS62740 (Texas Instruments)
- BLE SoC: nRF52832 (Nordic Semiconductor)
- ECG sensing unit: INA333 (Texas Instruments), MAX9638 (Maxim Integrated)
- Accelerometer unit: BMI160 (Bosch Sensortec)
- Thermometer unit: MAX30205 (Maxim Integrated)
- Limb Sensor
- Passive components: Capacitors, Inductors, Resistors; 0201 to 0402 footprint)
- Power management units: Bq25120a (Texas Instruments), TPS62740 (Texas Instruments)
- BLE Soc: nRF52832 (Nordic Semiconductor)
- PPG sensing unit: MAX30101 (Maxim Integrated)
- Thermometer unit: MAX30205 (Maxim Integrated)

*Sensor Disinfection Procedure*

The following describes a simple 10 step procedure to clean the device approved by the infection control committee of Lurie Children’s Hospital.

1. Wash your hands with soap and water before entering the patient’s room. Remove the sensors from the patient’s skin.
2. Dispose of all hydrogel adhesives in the trash can in the patient’s room.
3. Wipe all surfaces of the sensors with a PDI wipe continuously for 2 minutes.
4. Ensure that all surfaces of the sensors remain wet for full 2 minutes.
5. Allow the sensors to air dry completely.
6. Wipe all surfaces of the mobile device with a [PDI wipe](https://pdihc.com/products/environment-of-care/super-sani-cloth-germicidal-disposable-wipe/) (Sanicloth) continuously for 2 minutes.
7. Ensure that all surfaces of the computer remain wet for a full 2 minutes. Allow the study computer to air dry completely.
8. Before returning the sensors to their containers, inspect the disinfected sensors for visible breaks in the encasing.
9. If there is a visible break in a sensor’s encasing, place the sensor in a separate storage container, and return the sensor to the lab to be autoclaved and re-encased.
10. If there are no visible breaks in the sensor’s encasing, return the disinfected sensors to their clean container. Wash your hands with soap and water after exiting the patient’s room


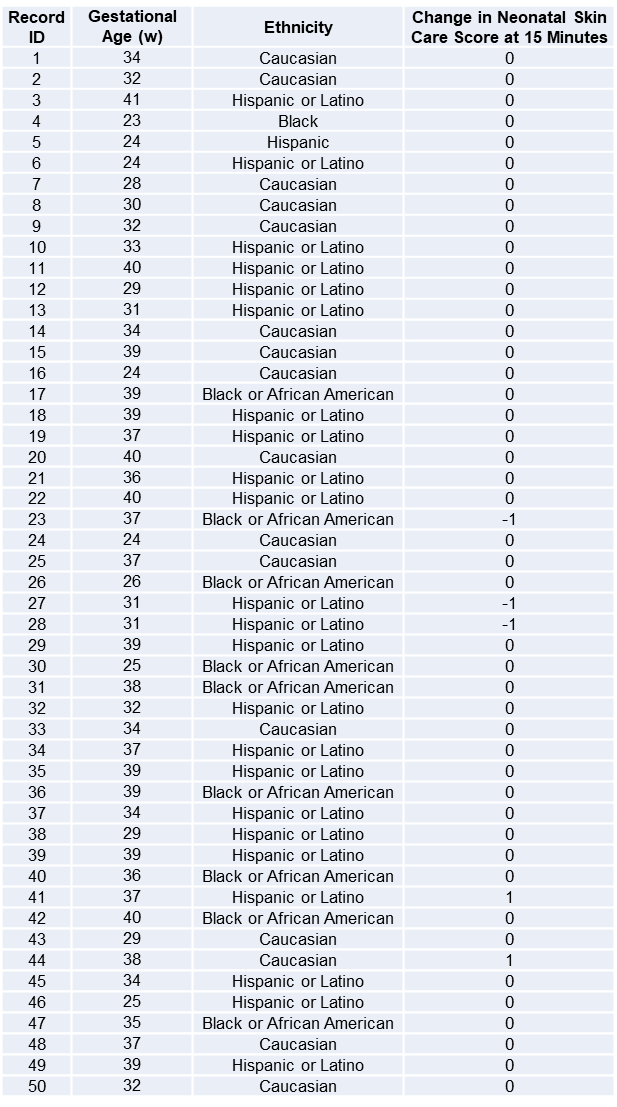


**Supplementary Table 1. Clinical characteristics of neonates admitted in the NICU/PICU in this study.** A change in skin score was determined using modified Neonatal Skin Condition Scale (3-9). The scale is used to score the underlying skin 15 minutes after removal of each sensor. The score is compared to the pre-testing skin. Higher scores indicate greater skin erythema (1-3), dryness (1-3), and breakdown (1-3). A perfect score is 3 where there is no evidence of skin dryness, erythema or breakdown. A score of 9 is the worst indicating very dry skin with cracking/fissures, visible erythema in > 50% of skin underneath the sensors, and extensive breakdown. The average change in the score (negative change suggests improvement) was -0.02. Only 2 subjects (4%) of subjects exhibited an increase in the scale, which was limited to a 1-point increase.


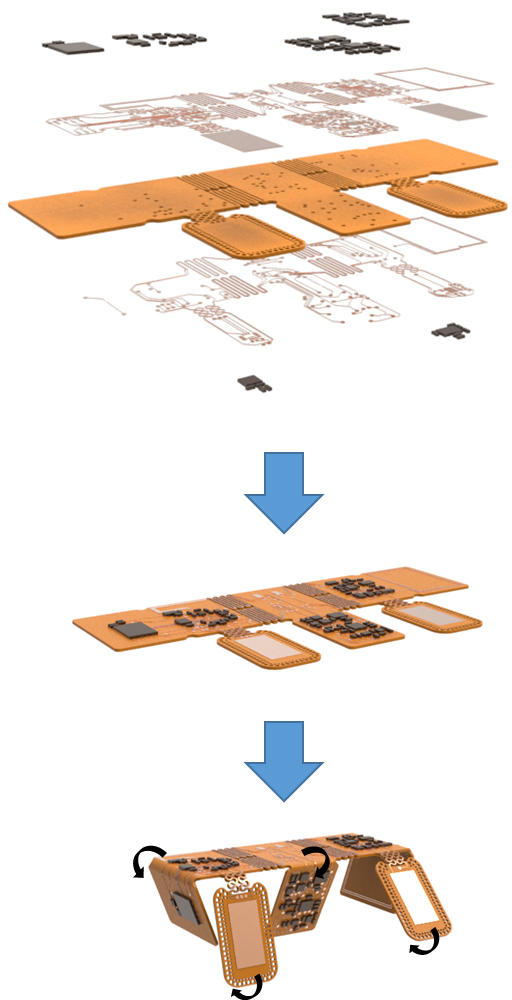


**Supplementary Fig. 1. The construction illustration of a chest unit.** After the 2-layer flexible printed circuit board is assembled with circuit components, sub-islands are folded.


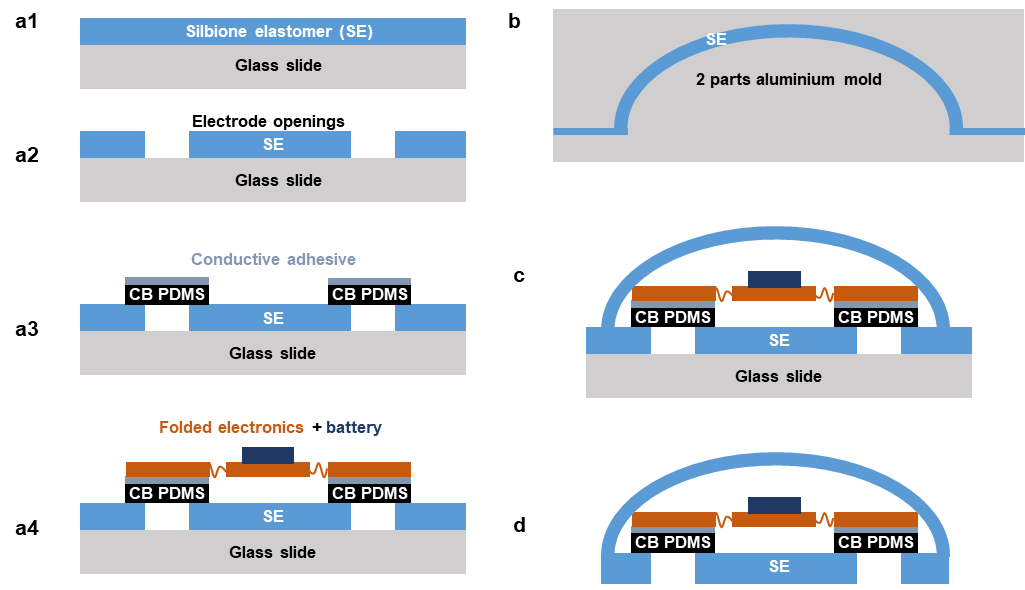


**Supplementary Fig. 2. Schematic summary of assembly steps for embedded chest device (as a representative example, some steps are simplified for visualization purposes) . a1,** Silbione RTV4420 elastomer spin coated on a glass slide (with a release layer not shown on schematic) yields a flat elastomer film. **a2,** Laser cutting provides openings. **a3,** Carbon Black PDMS and conductive adhesive adhered to Silbione elastomer provide a conductive sticky platform to receive the electronics. **a4,** Folding of electronics includes two copper pads attached to conductive adhesive**. b,** Molding yields a Silbione RTV4420 elastomer shell**. c,** Shell assembly to a4 step system thanks to uncured Silbione RTV4420 results in a sealed device. **d,** Edges are cut, and device is released from glass slide.


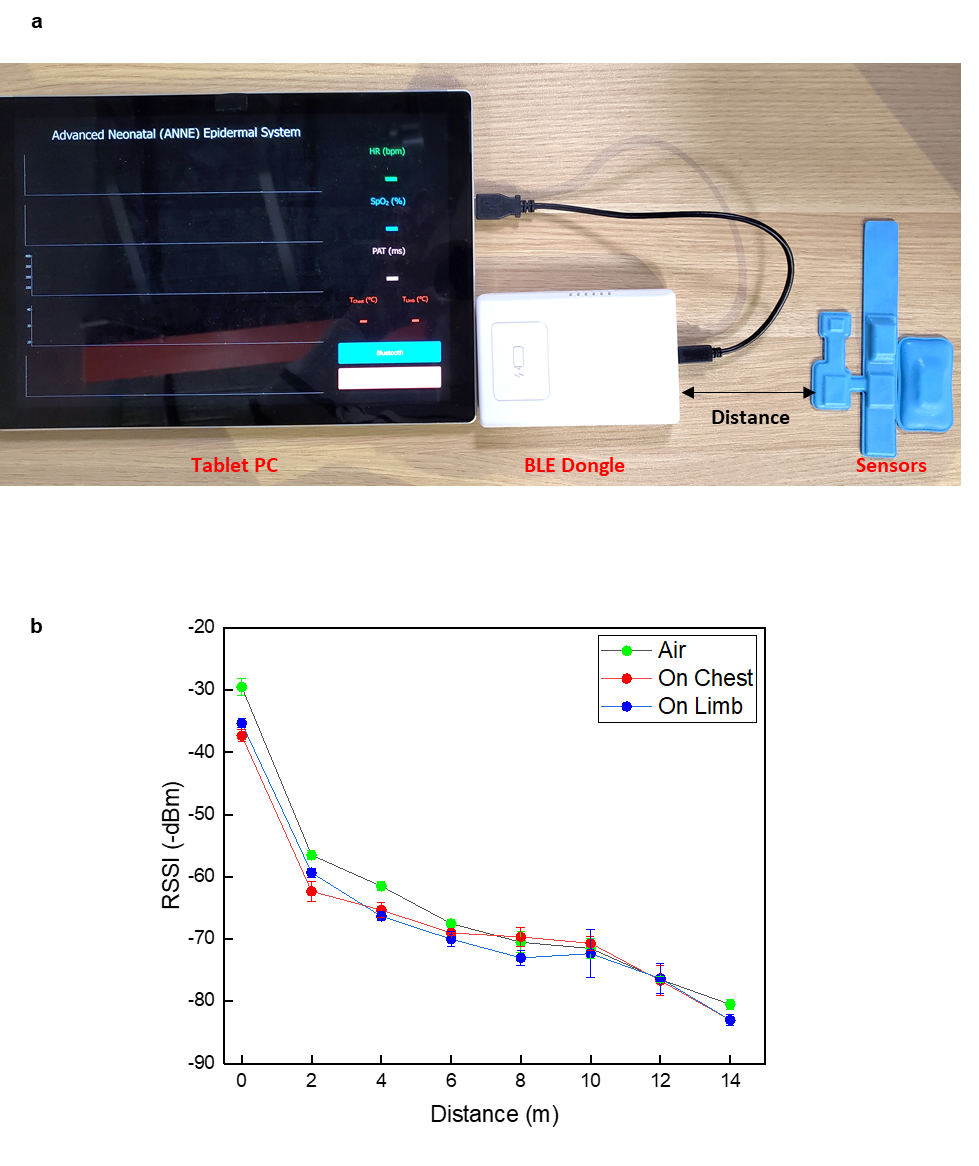


**Supplementary Fig. 3. Received Signal Strength Indicator (RSSI) of a chest and limb units. a,** Testing set up showing the connection of a BLE dongle to a tablet computer and sensors. RSSI is measured at every 2 meters between sensors and the BLE dongle without having obstructing objects in between. b, RSSI plot in three different cases (in the air, on chest, on limb). Error bars were obtained by repeating measurements for three times. RSSI indicates the wireless signal strength between a sensor and a BLE dongle (receiver), which demonstrates the sensor’s operating distance. RSSI level when sensors are in the air is slightly better compared when sensors are mounted on chest or limb. The signal strength is maintained to be robust up to 10 meters, which is sufficiently long enough for the robust wireless data transmission in a standard patient room.


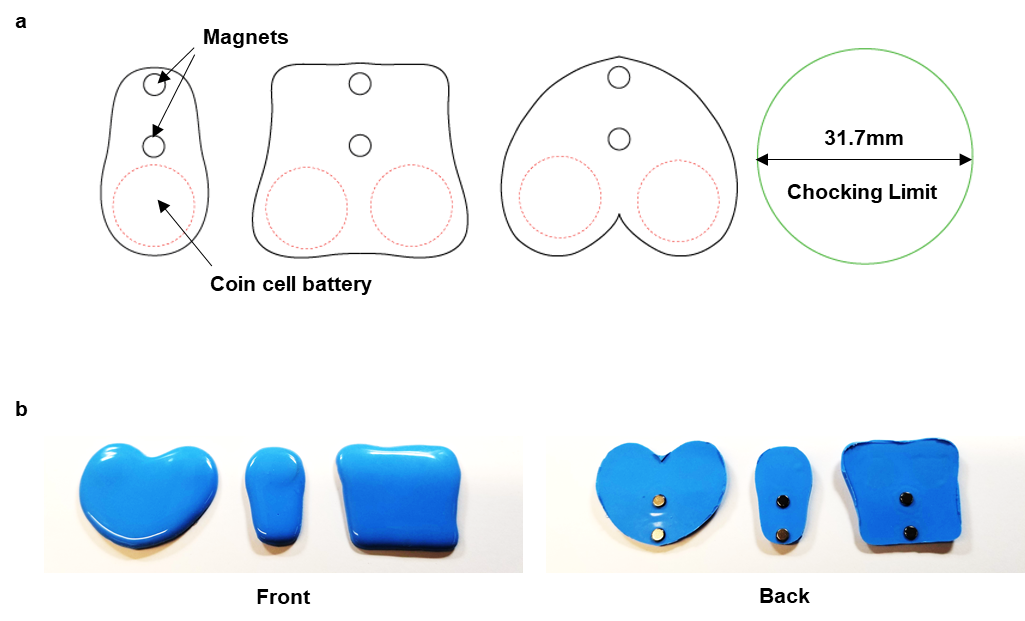


**Supplementary Fig. 4. Removable battery sizes options.** Modular batteries are encapsulated with various shapes, showing the possible compatibility with choking hazard prevention requirements. **a,** Schematic layouts highlighting position of magnets and of one- or two-coin cell batteries, and comparison with the 31.7mm diameter circle corresponding to choking hazard limit. **b,** Photographs of front side (left) and back side (right) of encapsulated batteries.


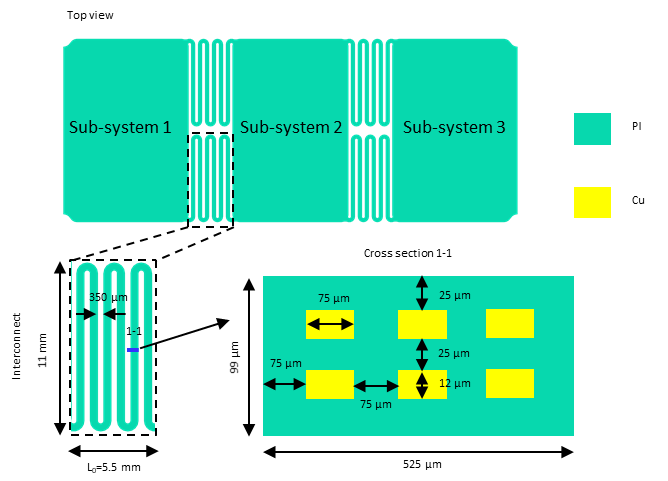


**Supplementary Fig. 5. Schematic illustration of the serpentine interconnects used in a chest unit.** The three sub-systems are linked mechanically and electrically by soft serpentine interconnects that provide high stretchability and conformably comply with physiological deformations when the device is mounted in the human body. The soft serpentine interconnects consist of two 12μm-thick copper layers encapsulated in polyimide (PI) and separated by 25 μm in the out-of-plane direction. Each copper layer features three serpentine traces with a width W = 75 μm and the in-plane separation between the traces is 75 μm. The total thickness of the serpentine interconnects is 99 μm.


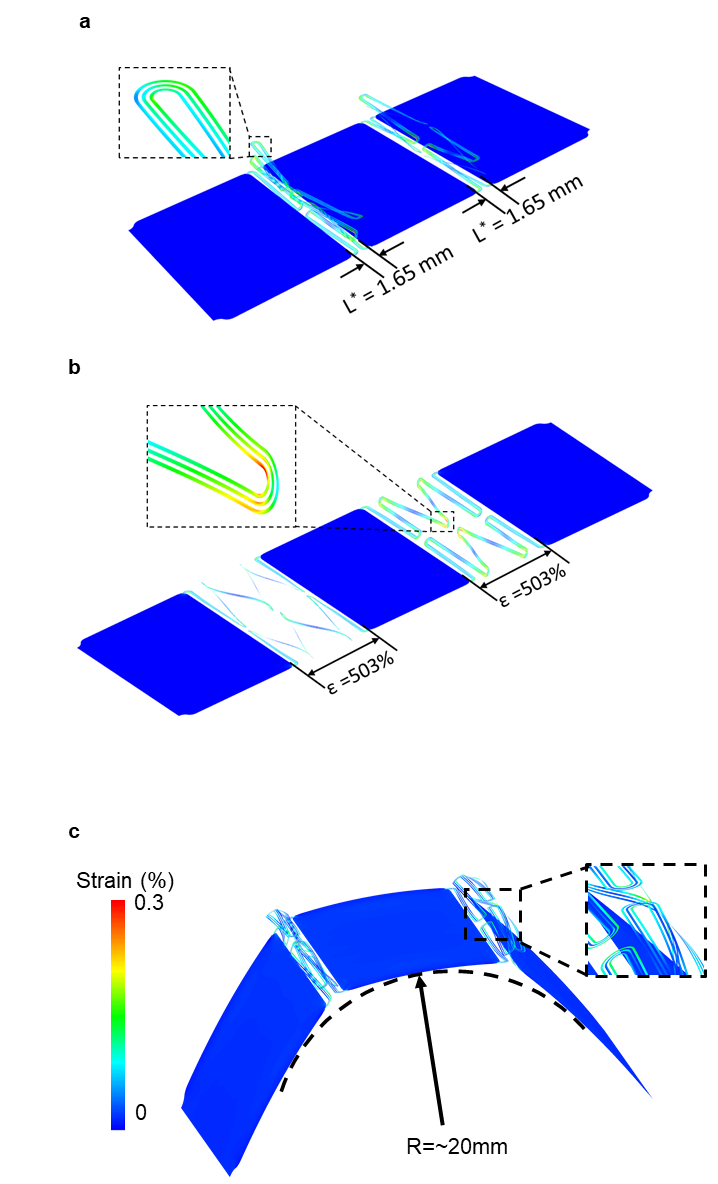


**Supplementary Fig. 6. Computational demonstration of the mechanical properties of a chest unit. a,** The initial length of interconnect (spacing between sub-systems) is L_0_ = 5 mm. To increase the elastic stretchability, the interconnect is pre-compressed such that its initial horizontal length is reduced from L_0_ = 5 mm mm to L* = 1.65 mm. **b,** The simulation results from the finite element analysis (FEA) indicate that the elastic stretchability of the designed and optimized interconnects achieves 503%. The elastic stretchability of the interconnects is defined as ε = (L-L*)/ L*, where L is the stretched length at which the copper layer in the interconnect yields. **c,** The simulation result of the strain in the copper layer of a chest unit for a bending radius of ~20 mm. The equivalent bending stiffness of the chest unit is ~9.6 Nmm^2^.


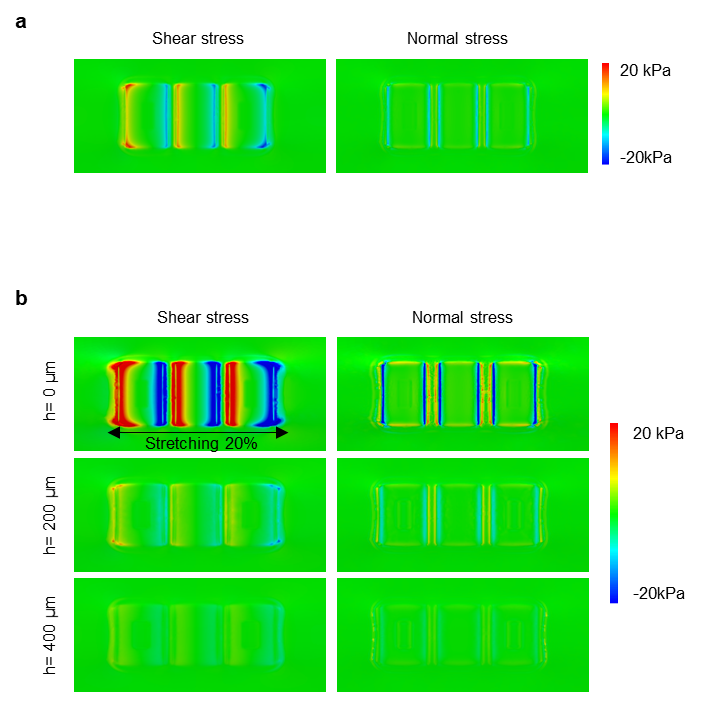


**Supplementary Fig. 7. Computation result of the effect of thickness of strain of isolation layer to shear and normal stress. a,** the stresses on the skin after the device without strain isolation layer is stretched by 6.5%. **b,** the stress distributions on the skin after the device with a strain isolation layer of different thicknesses (0, 200, and 400 µm) is stretched by 20%.


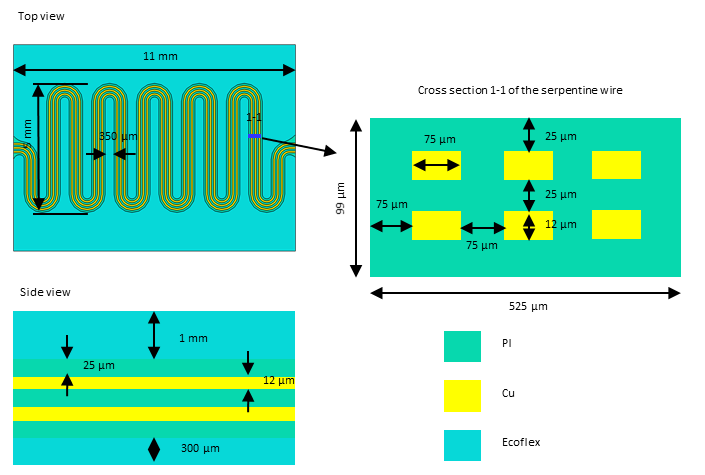


**Supplementary Fig. 8. Schematic illustration of a representative interconnects used in the limb unit.** The soft serpentine interconnects consist of two 12 μm-thick copper layers encapsulated in polyimide (PI) and separated by 25 μm in the out-of-plane direction. Each copper layer features three serpentine traces with a width W = 75 μm and the in-plane separation between the traces is 75 μm. The total thickness of the serpentine interconnects is 99 μm.


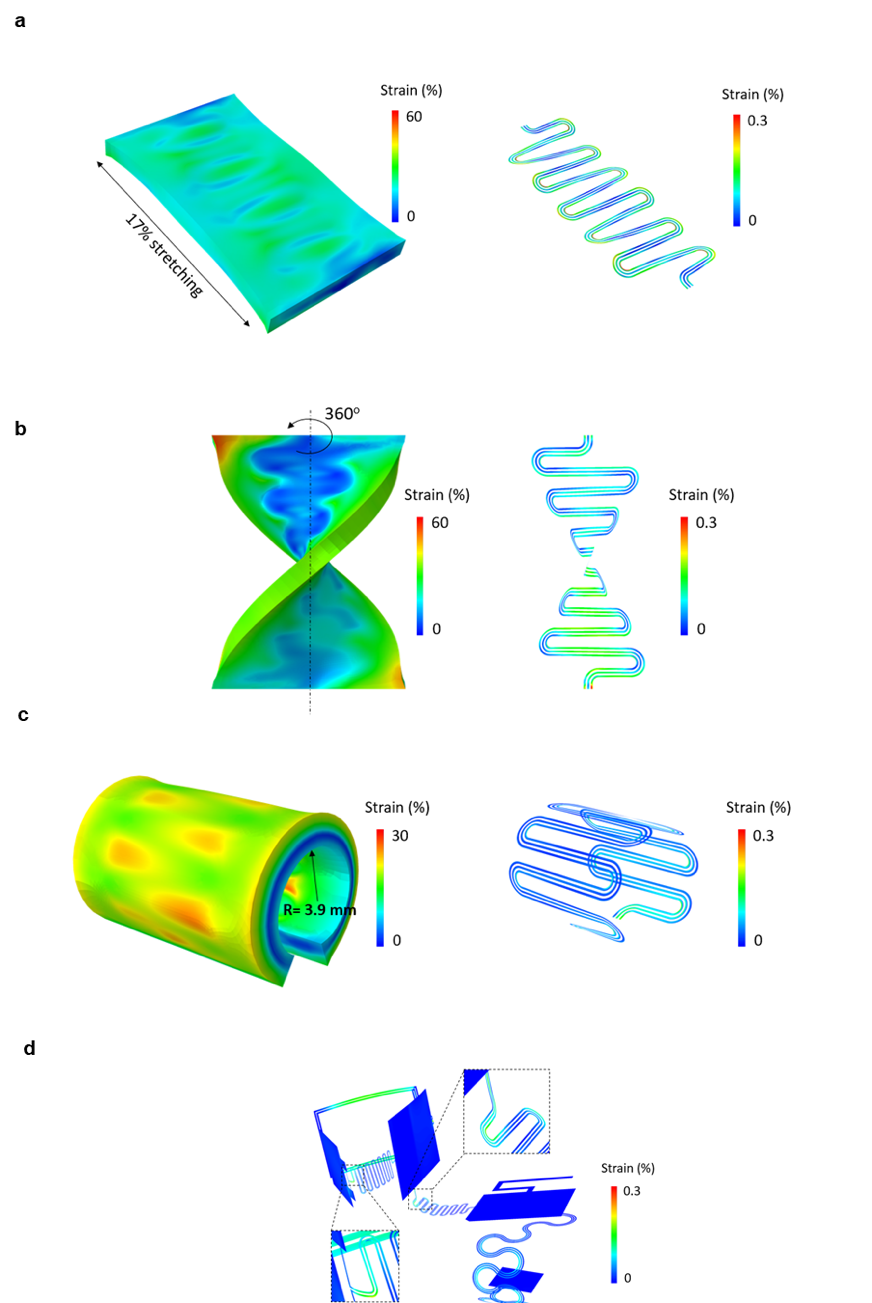


**Supplementary Fig. 9. Mechanical characteristics of a limb unit.** The strain distribution in the encapsulation layer (left), and copper layer (right) of a representative interconnect during **(a)** stretching, **(b)** twisting, **(c)** bending at the radius of 3.9 mm, and **(d)** the overall bending mechanics in a limb unit.


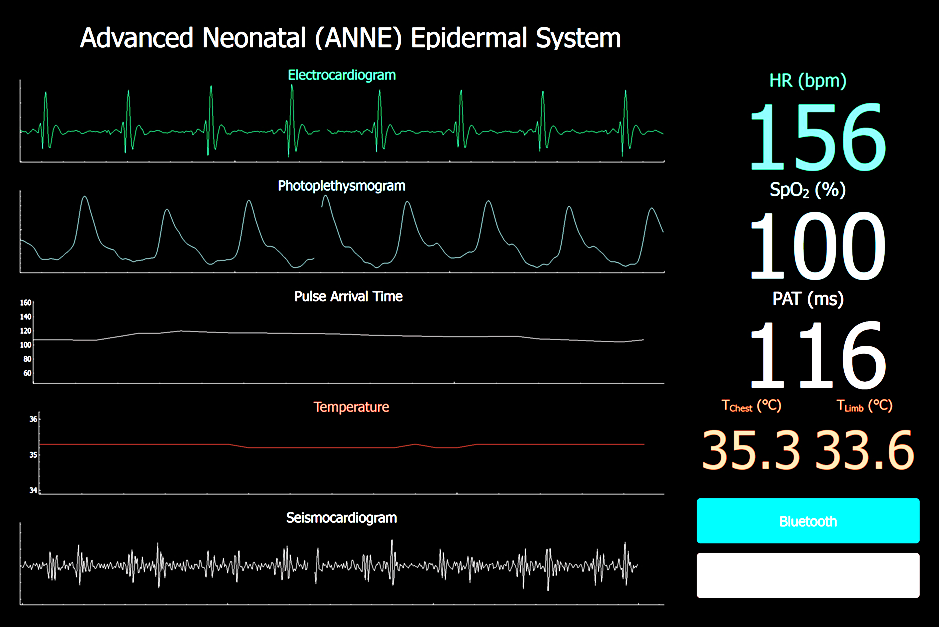


**Supplementary Fig 10. A Screenshot of the real-time waveform plotting and signal processing program.**


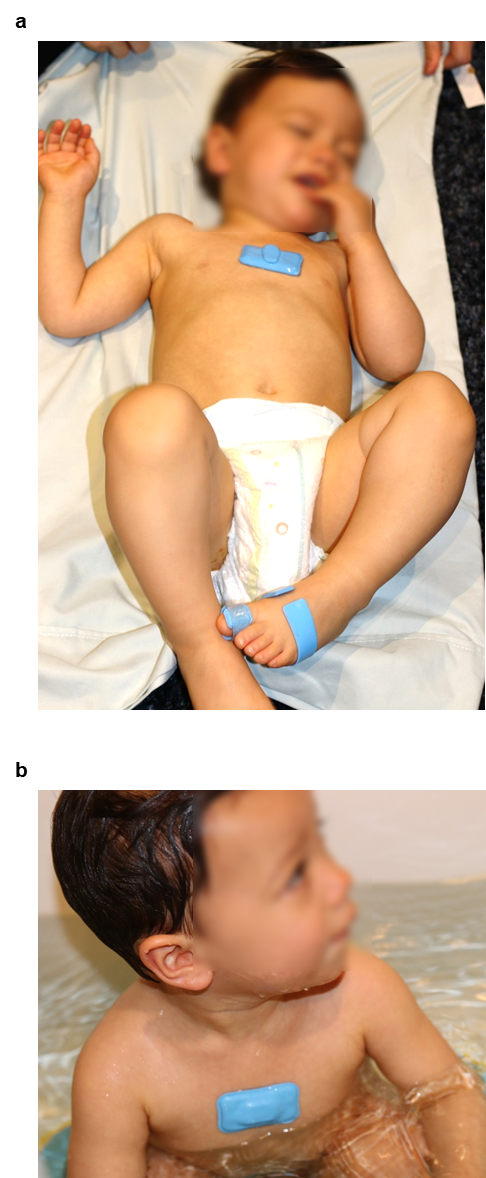


**Supplementary Fig. 11. Pictures of baby during hands-on care. a,** Change diaper. **b,** Bathe an infant.


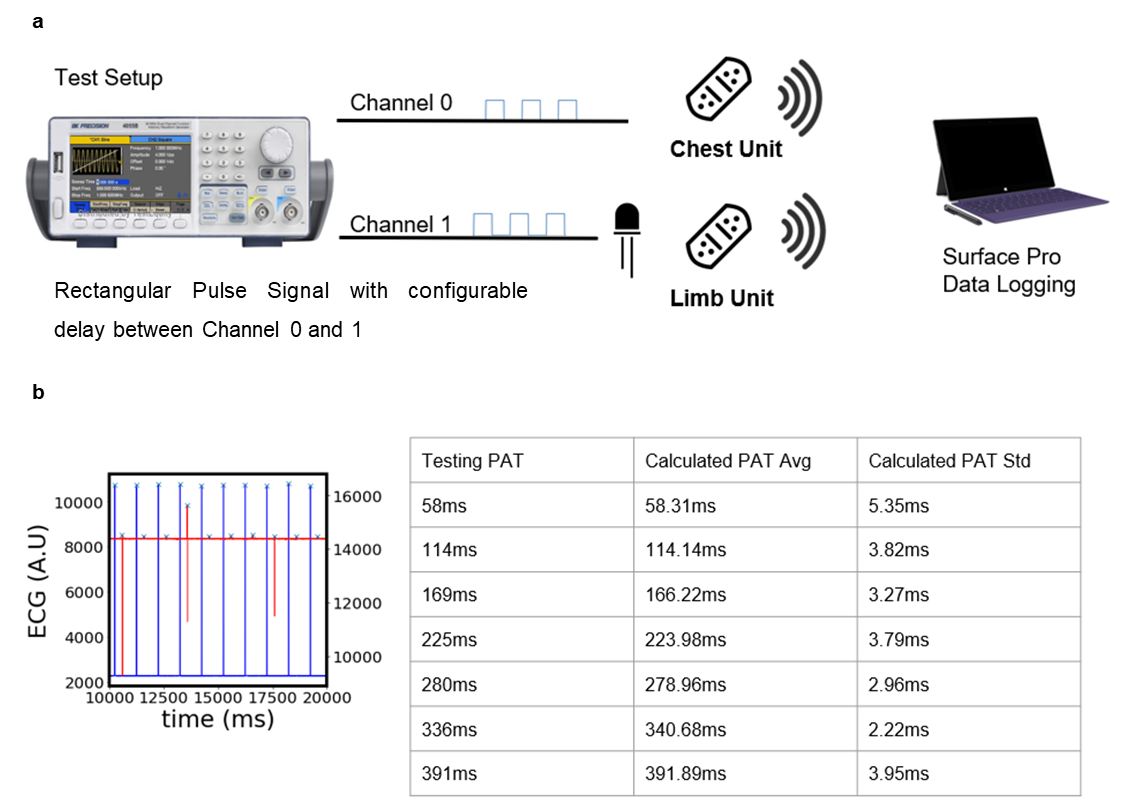


**Supplementary Fig. 12. Time-synchronization validation. a,** Signal generator creates rectangular pulse signal is fed into a chest unit and streamed to base station (Surface Pro). Delayed signal is fed into Red LED and the output of the photodetector is then processed in a limb unit and streamed to base station. **b,** The timing difference collected at each unit is compared by the means of average delay and standard deviation.


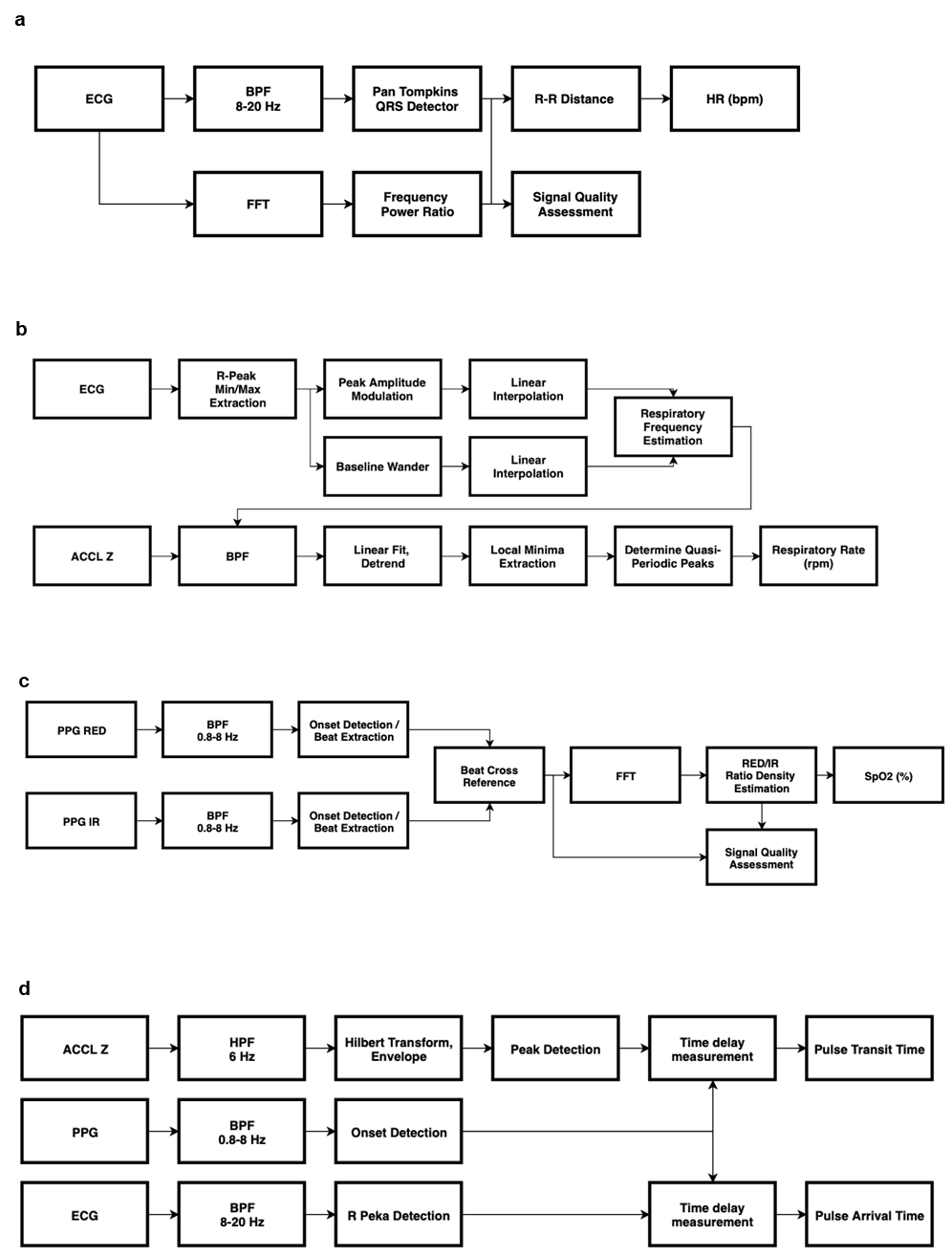


**Supplementary Fig. 13. Signal processing algorithm for heart rate (a), respiration rate (b), blood oxygenation (c), and pulse arrival and transit time (d).**


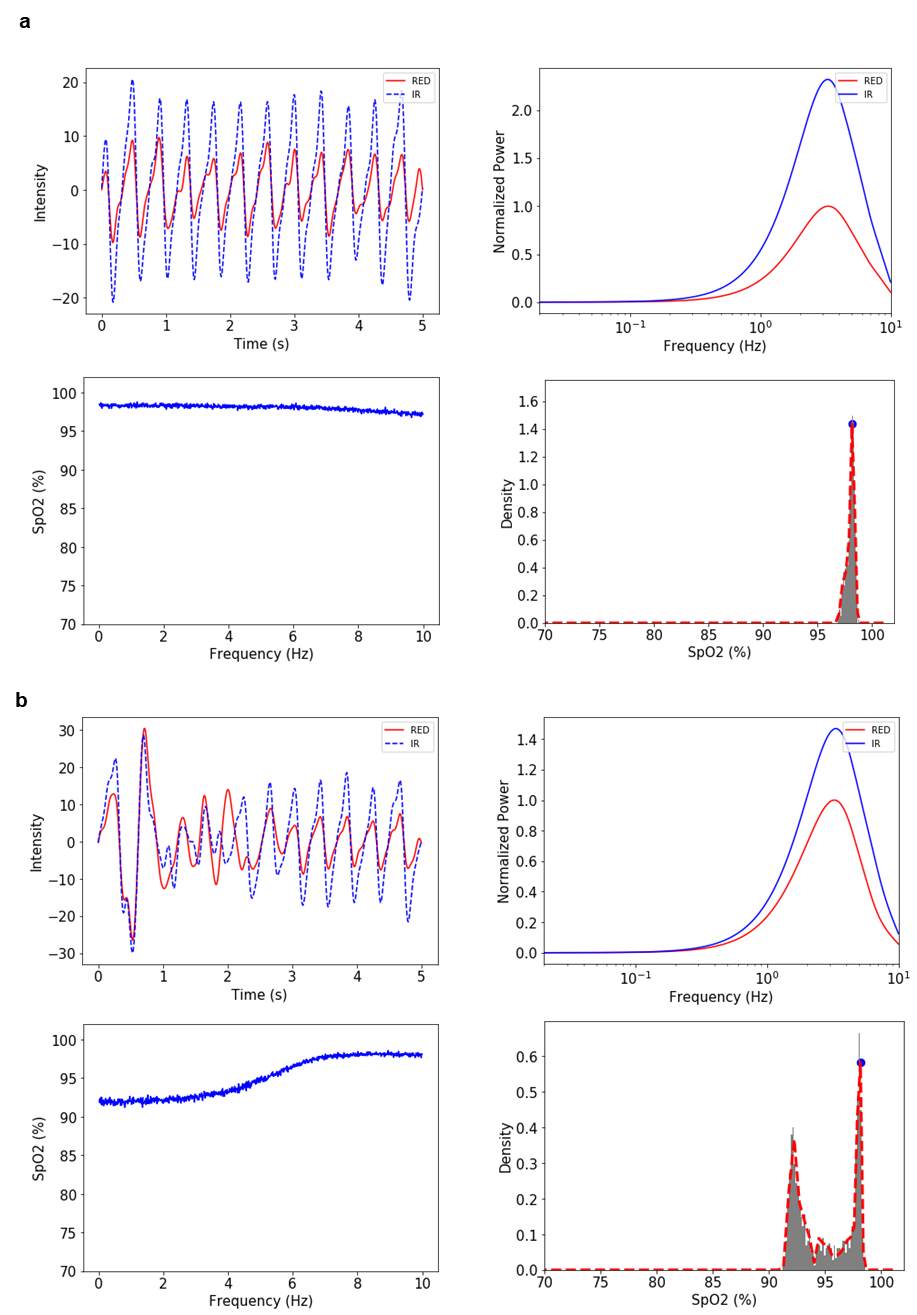


**Supplementary Fig. 14. Detailed signal processing algorithm for SpO_2_.** Processing of SpO2 calculation algorithm for the signal without motion artifact (a) and with motion artifact (b).


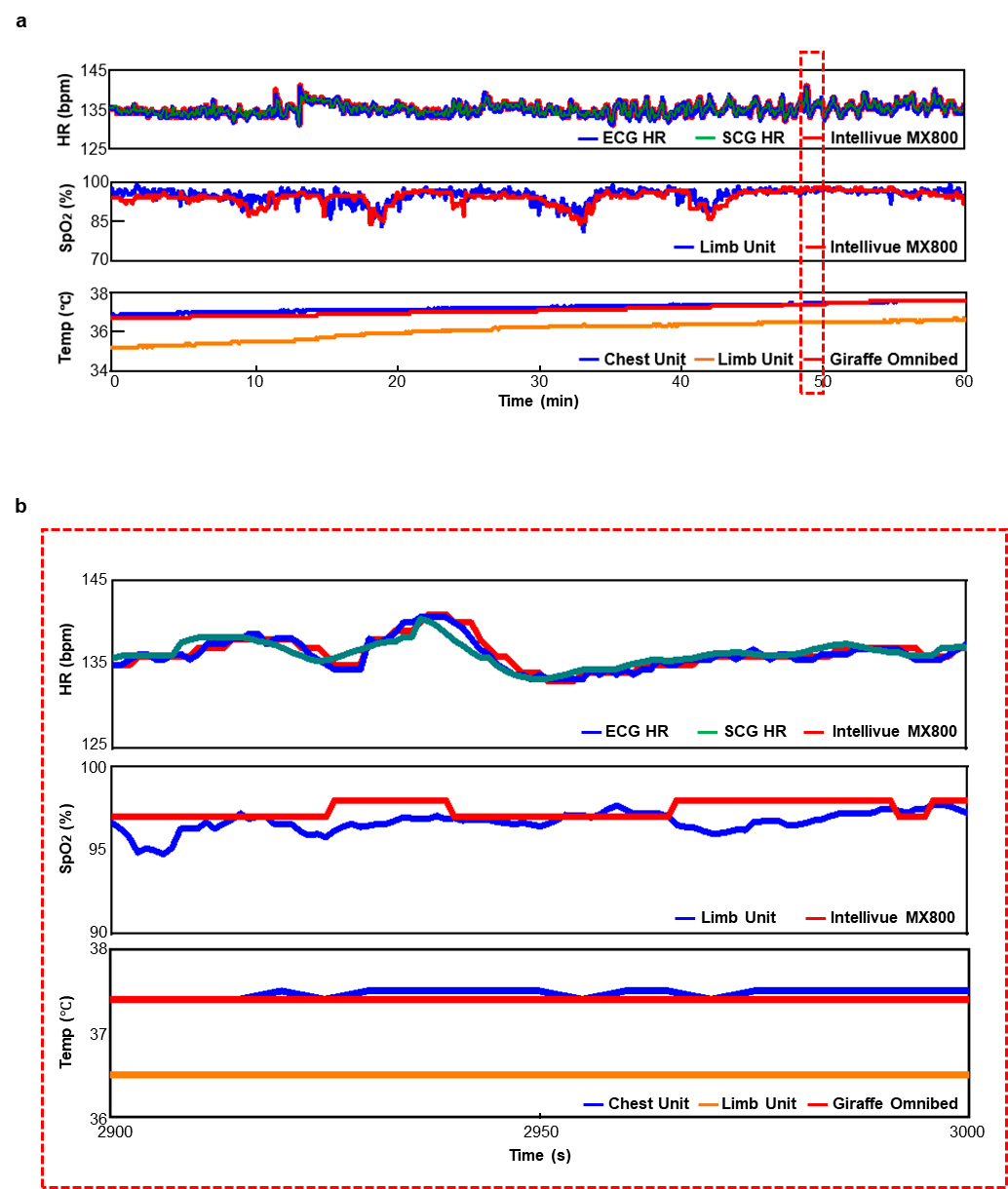


**Supplementary Fig. 15. A magnified plot from Fig. 4b. a.** Original plot of Fig. 4b. **b,** Magnified plot at around 50-minute.


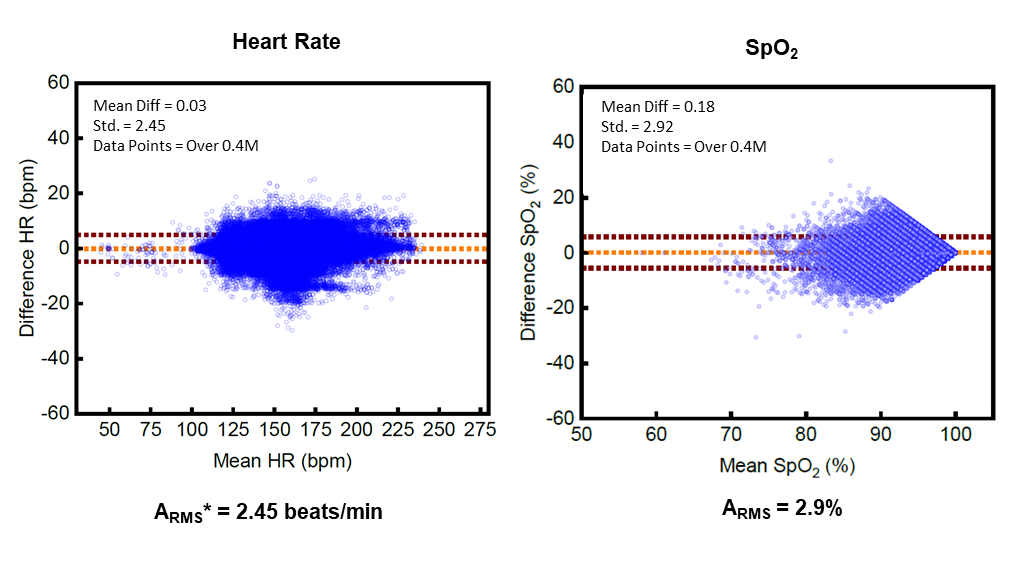


**Supplementary Fig. 16. The global BA plot for heart rate and blood oxygenation obtained in n = 20 (over 0.4 M data points).**


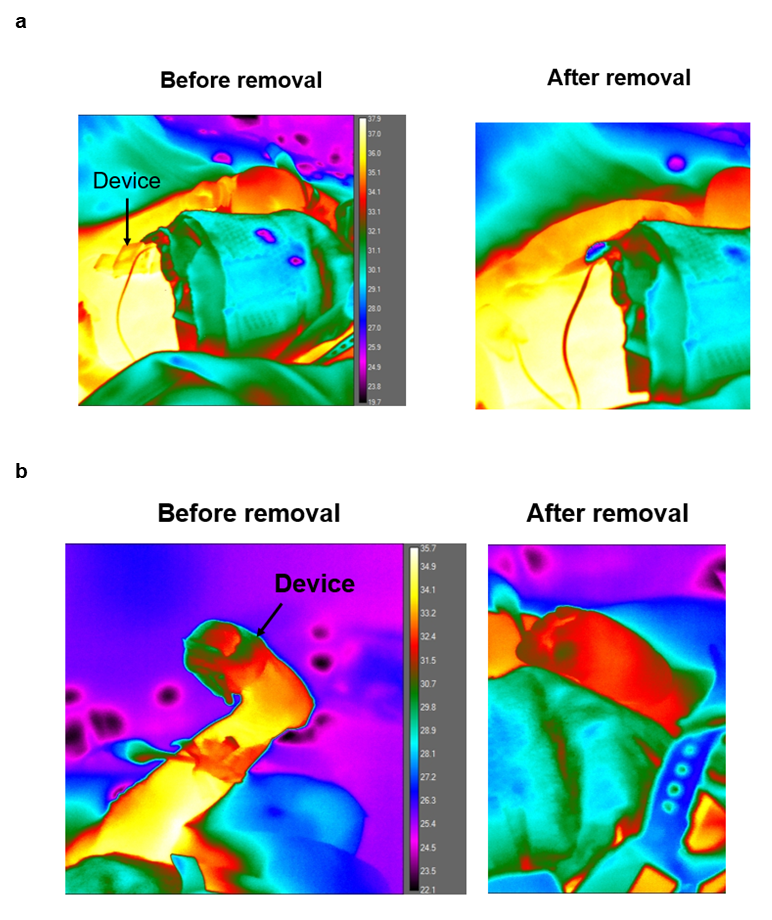


**Supplementary Fig. 17. The representative figures for safety related to heat generation of the device during 24-hr operation. a,** A chest unit did not create any significant heating after 24-hr operation. **b,** A limb unit did not create any significant heating after 24-hr operation.


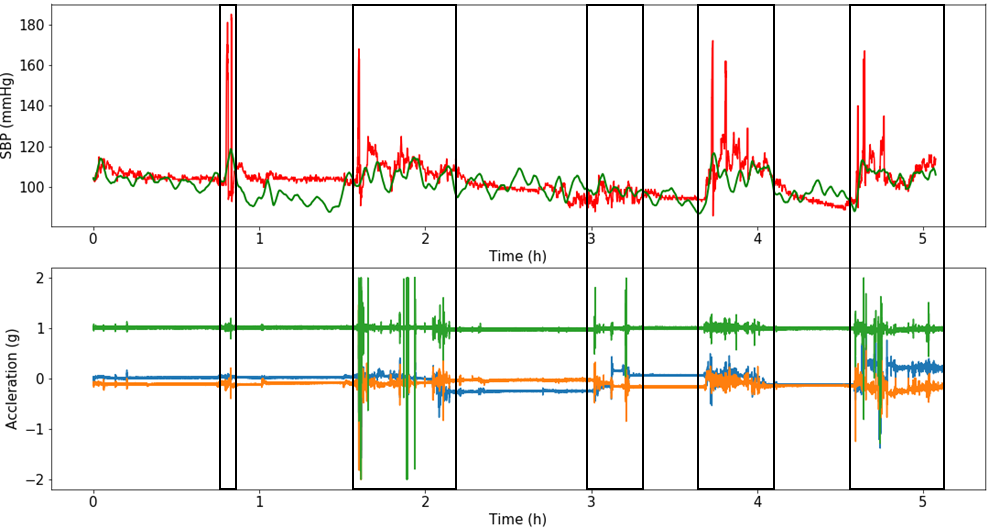


**Supplementary S18. Capturing the events with motion artifact by the accelerometry data in a chest unit.** Observation of larger movement in accelerometry data suggests that the spikes in SBP measured by A-line (red color) has a direct effect from motion of a subject.


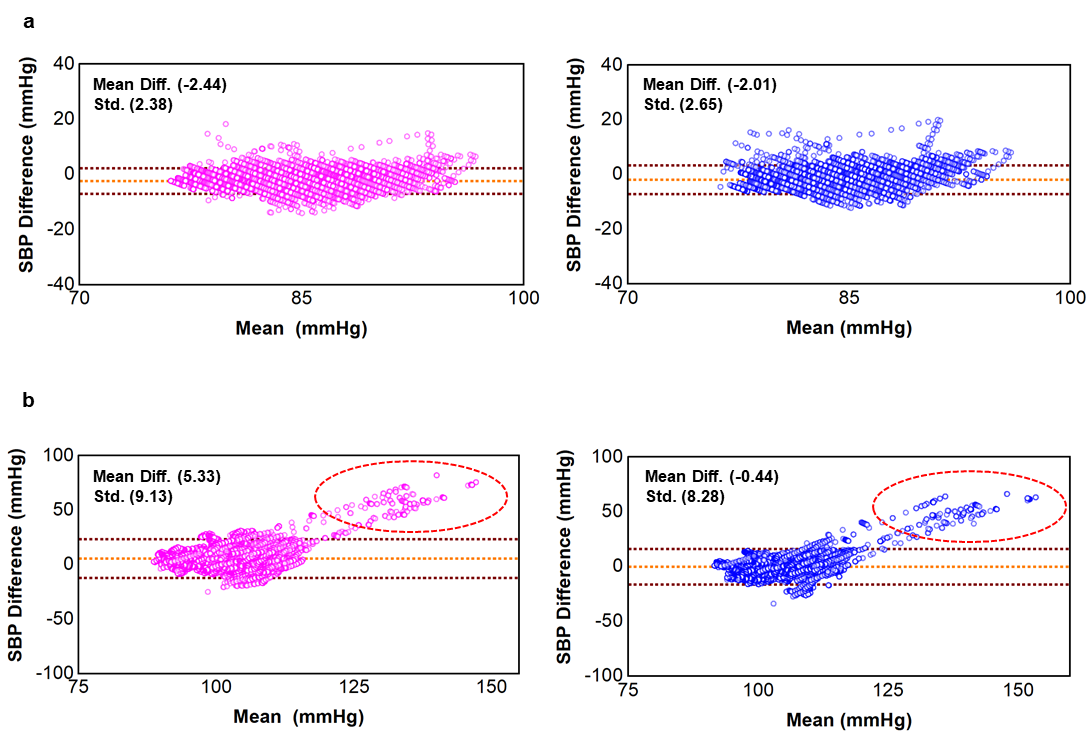


**Supplementary Fig. 19. Individual Bland Altman Plot of data presented in Fig. 5. a,** Bland Altman plot of SBP A-line vs SBP A-line PAT (left) and SBP A-line vs SBP PTT (right) obtained from a subject with a condition of acute hypoxemic respiratory failure, rhinovirus and coronavirus infection (40 w GA, 50 w CA). **b,** Bland Altman Plot of SBP A-line vs SBP A-line PAT (left) and SBP A-line vs SBP PTT (right) obtained from a subject with a condition of chronic kidney disease (hypovolemia dehydration), Wolf-Hirschhorn syndrome, and acute respiratory failure with hypoxia and hypercapnia (34 w GA, 40 w CA). Standard deviation above 8 mmHg is largely affected by the circled regions in red where A-line experienced large jumps in blood pressure and our accelerometry data suggested motion artifact associated with these jumps (Supplementary Fig. 18).


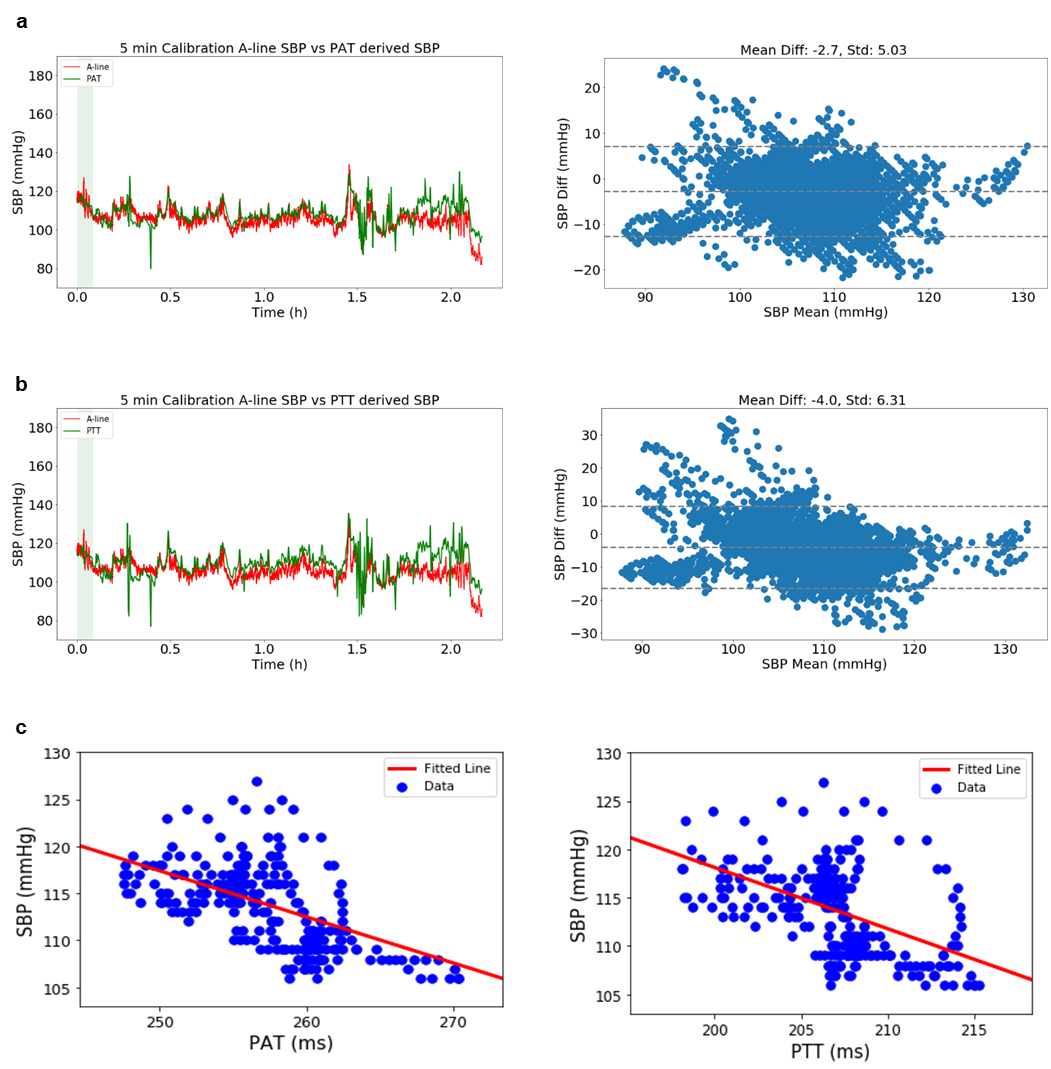


**Supplementary Fig. 20. Blood pressure analysis on a subject with H/O splenectomy (39 w GA, 95 w CA) at first session. a,** A plot of SBP A-line (red) and SBP PAT (green) for ~2 h of data collection (left) and resulting Bland Altman Plot (right). **b,** A plot of SBP A-line (red) and SBP PTT (green) for ~2 h of data collection (left) and resulting Bland Altman plot (right). **c,** 5-minute of calibration plot for PAT (left) and PTT (right).


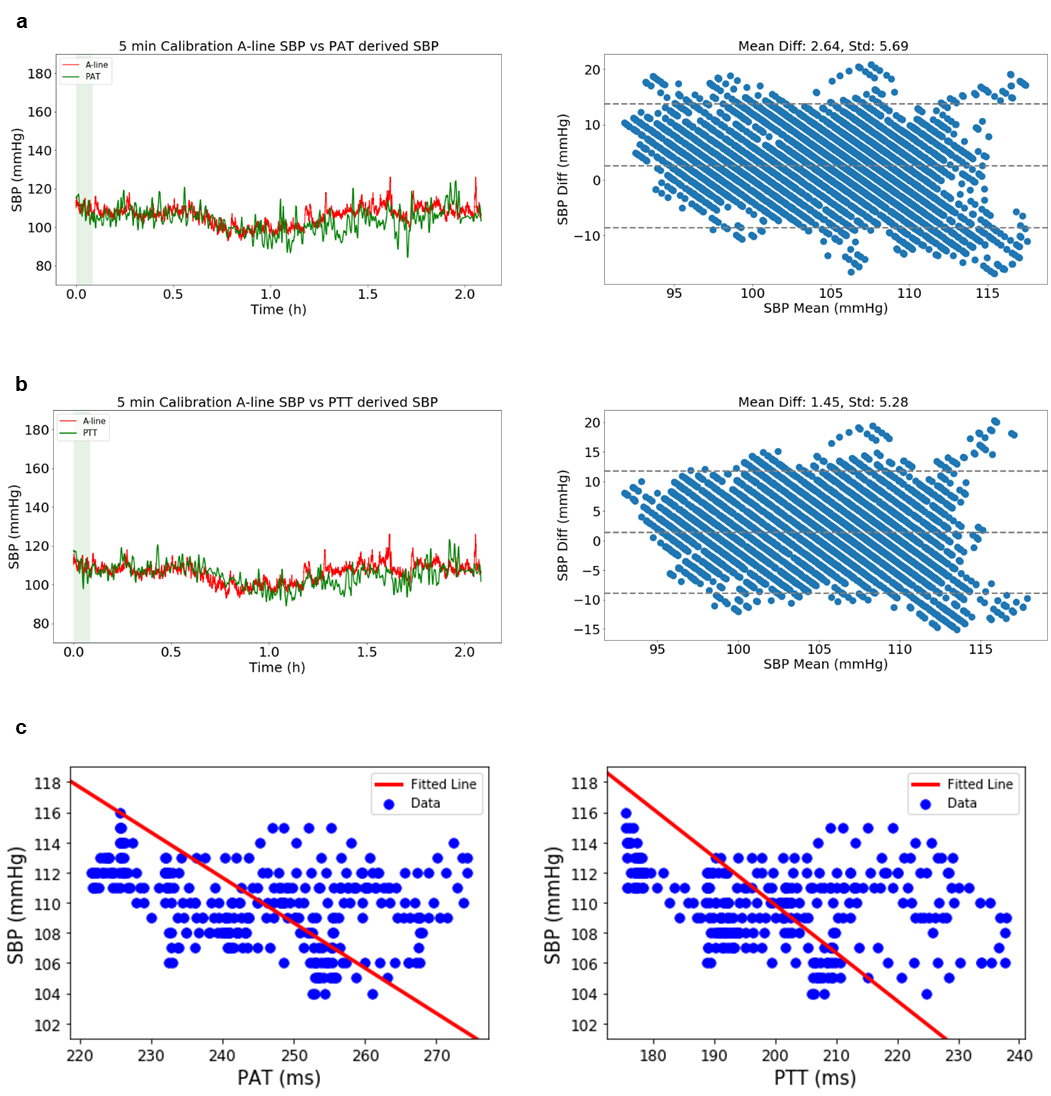


**Supplementary Fig. 21. Blood pressure analysis on a subject with H/O splenectomy (39 w GA, 95 w CA) at second session after 30 minutes of first session. a,** A plot of SBP A-line (red) and SBP PAT (green) for ~2 h of data collection (left) and resulting Bland Altman Plot (right). **b,** A plot of SBP A-line (red) and SBP PTT (green) for ~2 h of data collection (left) and resulting Bland Altman plot (right). **c,** 5-minute of calibration plot for PAT (left) and PTT (right).


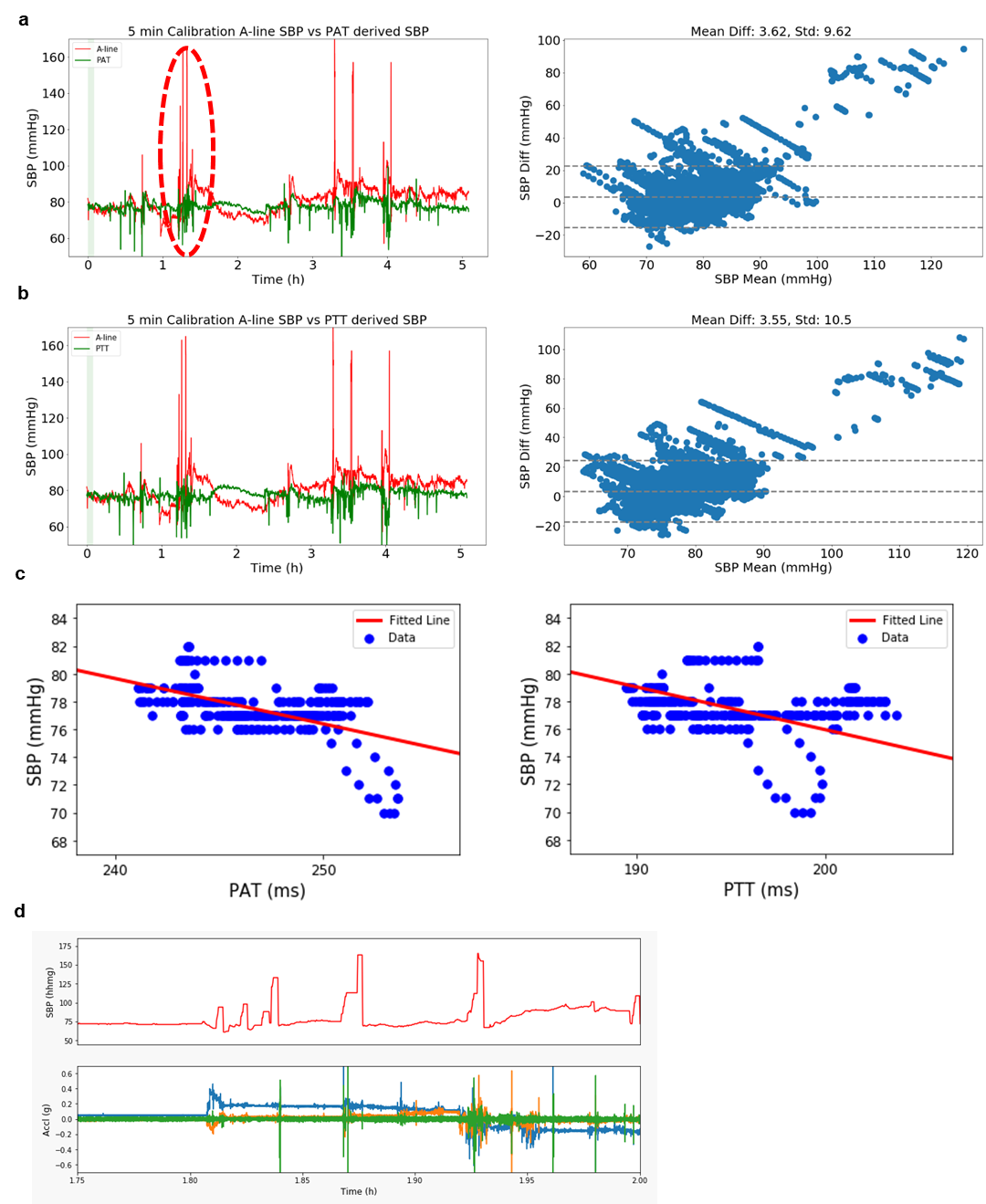


**Supplementary Fig. 22. Blood pressure analysis on a subject with acute respiratory failure with hypoxia and hypercapnia (40 w GA, 69 w CA). a,** A plot of SBP A-line (red) and SBP PAT (green) for ~5 h of data collection (left) and resulting Bland Altman Plot (right). **b,** A plot of SBP A-line (red) and SBP PTT (green) for ~5 h of data collection (left) and resulting Bland Altman plot (right). **c,** 5-minute of calibration plot for PAT (left) and PTT (right). **d,** A zoom-in plot of circled part in a, to demonstrate the effect of motion artifact associated at unusual spikes in A-line SBP data.


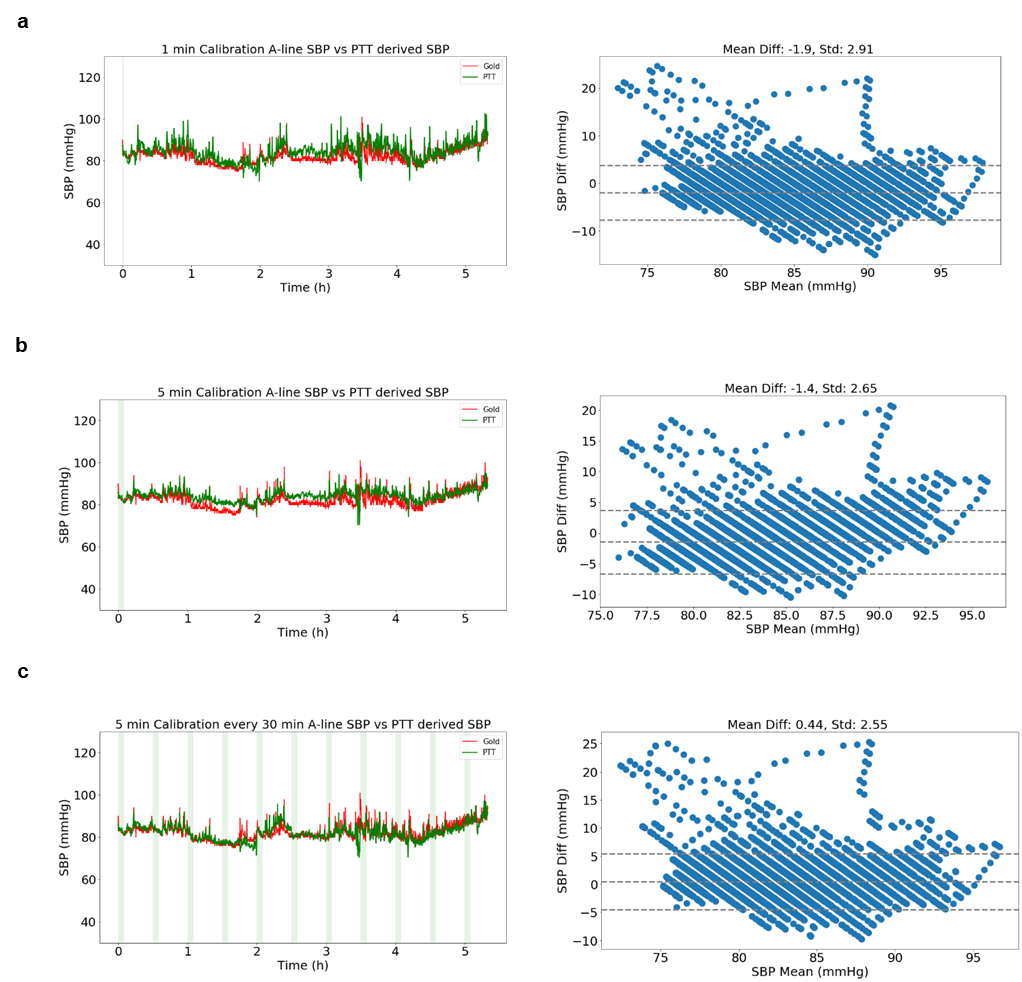


**Supplementary Fig. 23. The effect of calibration of window size and re-calibration interval. a,** Single calibration takes place with the initial one minute and **b,** five minutes of PT data against A-line. **c,** Another calibration scheme involves with re-calibration at every 30 minutes with the duration of 5 minutes of data. Longer duration of calibration shows the improvement both in mean difference and standard deviation. Re-calibration shows the effect in reducing mean difference.


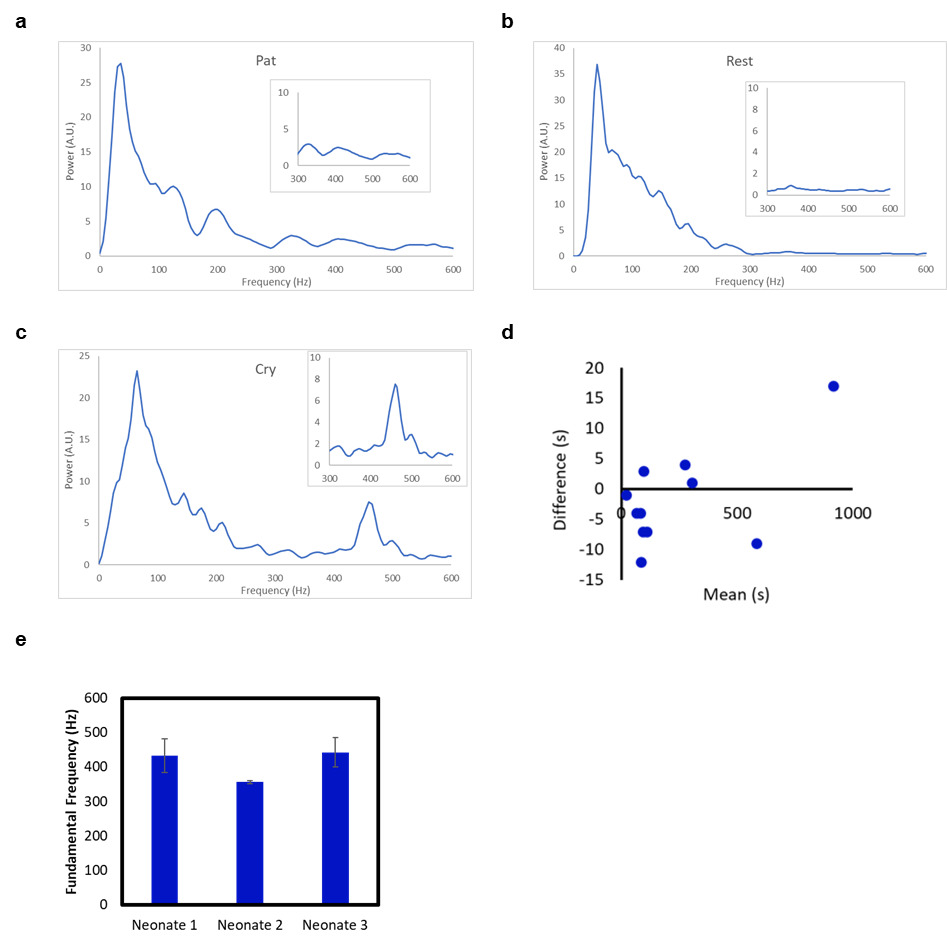


**Supplementary Fig. 24. Cry characteristics captured by a chest unit in NICU**. **a-c,** Representative power spectrum of signal frequency upon fast Fourier transform processing of neonatal mechano-acoustic signal during crying and non-crying events from a neonate in NICU. Neonatal mechano-acoustic signal is presented from **(a)** parent patting, **(b)** resting events, and **(c)** neonatal crying. **d,** Comparison of cry duration analysis between a chest unit and human recording of individual cry events. **f,** Fundamental frequency of cry from each neonate (n = 3).


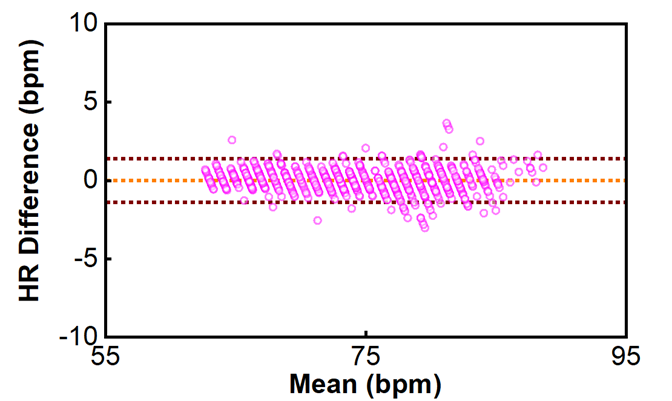


**Supplementary Fig. 25. The global BA plot for heart rate and blood oxygenation obtained in the all population (over 0.4 M data points).**
